# Myeloid-mesenchymal crosstalk drives ARG1-dependent profibrotic metabolism via ornithine in lung fibrosis

**DOI:** 10.1172/JCI188734

**Published:** 2025-08-28

**Authors:** Preeti Yadav, Javier Gómez Ortega, Prerna Dabral, Whitney Tamaki, Charles Chien, Kai-Chun Chang, Nivedita Biswas, Sixuan Pan, Julia Nilsson, Xiaoyang Yin, Aritra Bhattacharyya, Kaveh Boostanpour, Tanay Jujaray, Jasper T. Wang, Tatsuya Tsukui, Christopher J. Molina, Vincent C. Auyeung, Dean Sheppard, Baosheng Li, Mazharul Maishan, Hiroki Taenaka, Michael A. Matthay, Rieko Muramatsu, Lenka Maliskova, Arnab Ghosh, Walter L. Eckalbar, Ari B. Molofsky, Stanley J. Tamaki, Trever G. Bivona, Adam R. Abate, Allon Wagner, Satish K. Pillai, Paul J. Wolters, Kevin M. Tharp, Mallar Bhattacharya

**Affiliations:** 1 Division of Pulmonary, Critical Care, Allergy, and Sleep, Department of Medicine,; 2 Sandler Asthma Basic Research Center, and; 3 Department of Medicine, UCSF, San Francisco, California, USA.; 4 Vitalant Research Institute, San Francisco, California, USA.; 5 Department of Laboratory Medicine, and; 6 Helen Diller Family Comprehensive Cancer Center, UCSF, San Francisco, California, USA.; 7 Department of Electrical Engineering and Computer Sciences, and; 8 Center for Computational Biology, University of California, Berkeley, California, USA.; 9 Twist Bioscience, South San Francisco, California, USA.; 10 Department of Bioengineering and Therapeutic Sciences, UCSF, San Francisco, California, USA.; 11 Department of Radiation Oncology, Shandong Cancer Hospital and Institute, Shandong First Medical University and Shandong Academy of Medical Sciences, Jinan, China.; 12 Biological Science Division, Indian Statistical Institute, Kolkata, India.; 13 Cardiovascular Research Institute, UCSF, San Francisco, California, USA.; 14 Department of Molecular Pharmacology, National Institute of Neuroscience, National Center of Neurology and Psychiatry, Kodaira, Japan.; 15 UCSF CoLabs, UCSF, San Francisco, California, USA.; 16 Chan-Zuckerberg Biohub, San Francisco, California, USA.; 17 Department of Molecular and Cell Biology, University of California, Berkeley, California, USA.; 18 Cancer Metabolism and Microenvironment Program, National Cancer Institute–Designated Cancer Center, Sanford Burnham Prebys Medical Discovery Institute, La Jolla, California, USA.

**Keywords:** Immunology, Pulmonology, Amino acid metabolism, Fibrosis

## Abstract

Idiopathic pulmonary fibrosis (IPF) is a disease of progressive lung remodeling and collagen deposition that leads to respiratory failure. Myeloid cells are abundant in IPF lung and in murine lung fibrosis, but their functional effects are incompletely understood. Using mouse and human lung models, we show that ornithine produced by myeloid cells expressing arginase 1 (ARG1) serves as a substrate for proline and collagen synthesis by lung fibroblasts. The predominant ARG1-expressing myeloid cells in mouse lung were macrophages, but in IPF lung, high-dimensional imaging revealed ARG1 was expressed mainly in neutrophils. Small-molecule ARG1 inhibition suppressed both ornithine levels and collagen expression in cultured, precision-cut IPF lung slices and in murine lung fibrosis. These results were confirmed in macrophage-specific *Arg1*-KO mice. Furthermore, we found that this pathway is regulated by cell-to-cell crosstalk, starting with purinergic signaling: extracellular ATP receptor P2RX4 was necessary for fibroblast IL-6 expression, which, in turn, was necessary for ARG1 expression by myeloid cells. Taken together, our findings define an immune-mesenchymal circuit that governs profibrotic metabolism in lung fibrosis.

## Abstract 2

Crosstalk between myeloid cells and fibroblasts was found to increase ARG1 and hence levels of ornithine, which induced pathologic collagen expression in lung fibrosis.

## Graphical abstract

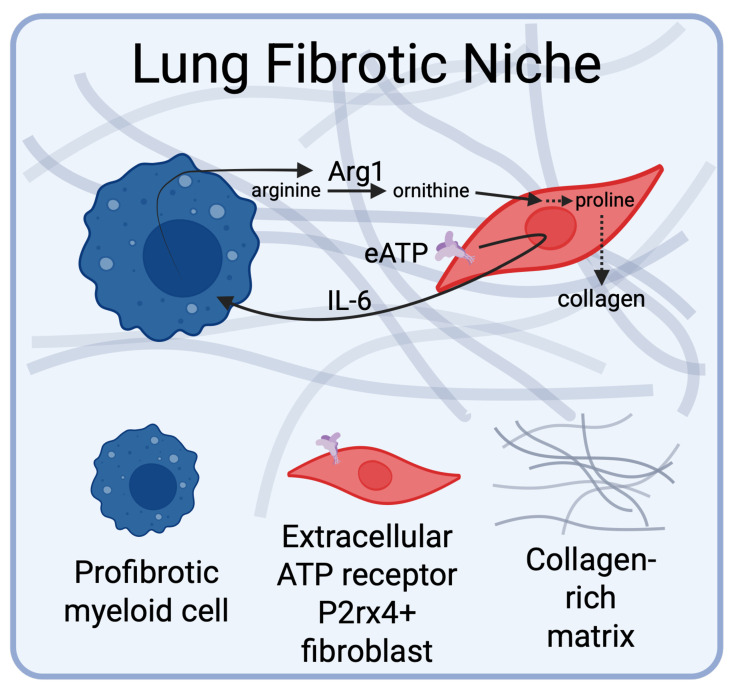

## Introduction

In idiopathic pulmonary fibrosis (IPF), epithelial dysfunction leads to the recruitment and activation of multiple cell types, including fibroblasts and myeloid cells (1). These populations have been found to be in close proximity to each other (2, 3), but it remains unclear how they interact to induce the fibrotic phenotype. One approach is to test the functional effects of diffusible factors in cell-to-cell communication. We recently considered the role of extracellular ATP (eATP), a damage-associated molecular pattern (DAMP) molecule, levels of which were found to be elevated in IPF lung (4). We found that fibroblast-specific deletion of the eATP receptor P2rx4 decreased lung fibrosis in mice (5). Here, we explored the effect of fibroblast P2rx4 signaling on cellular crosstalk in the lung fibrotic niche.

Single-cell RNA-Seq (scRNA-Seq) analysis of murine macrophage-fibroblast cocultures revealed that macrophage *Arg1* expression was decreased by deletion of *P2rx4* in cocultured fibroblasts. Numbers of *Arg1*^+^ macrophages were increased in murine lung fibrosis and localized in proximity to fibroblasts in areas of fibrosis. In IPF lung, numbers of ARG1-expressing cells were likewise increased but were predominantly neutrophils. In both murine lung fibrosis and cultured, precision-cut IPF lung slices, ARG1 inhibition decreased collagen expression. We then found in both mouse and human systems that ARG1 was induced by fibroblast IL-6 in a P2RX4-dependent manner.

Mechanistically, ARG1 drove fibroblast collagen synthesis via the production of ornithine, which served as a precursor for the collagen building block proline. These findings indicate a critical profibrotic role for a metabolic pathway arising from cellular crosstalk in the development of lung fibrosis.

## Results

### ARG1^+^ cells localize to the fibrotic niche.

To screen for macrophage genes regulated by fibroblast eATP/P2RX4 signaling, we used an in vitro system: either WT or *P2rx4*-KO fibroblasts were cocultured with WT lung macrophages (Supplemental Figure 1A; supplemental material available online with this article; https://doi.org/10.1172/JCI188734DS1), followed by scRNA-Seq. Both cell types were isolated from murine lungs 7 days after injury to approximate cellular states during the early fibrotic period, which was defined by increased fibroblast expression of *Col1a1* and *Col3a1* (Supplemental Figure 1B). Fibroblasts were used directly, whereas macrophages were treated for 48 hours in culture with CSF1 to maintain macrophage identity prior to coculture. After 5 days of coculture, cells were submitted for scRNA-Seq.

**Figure 1:**
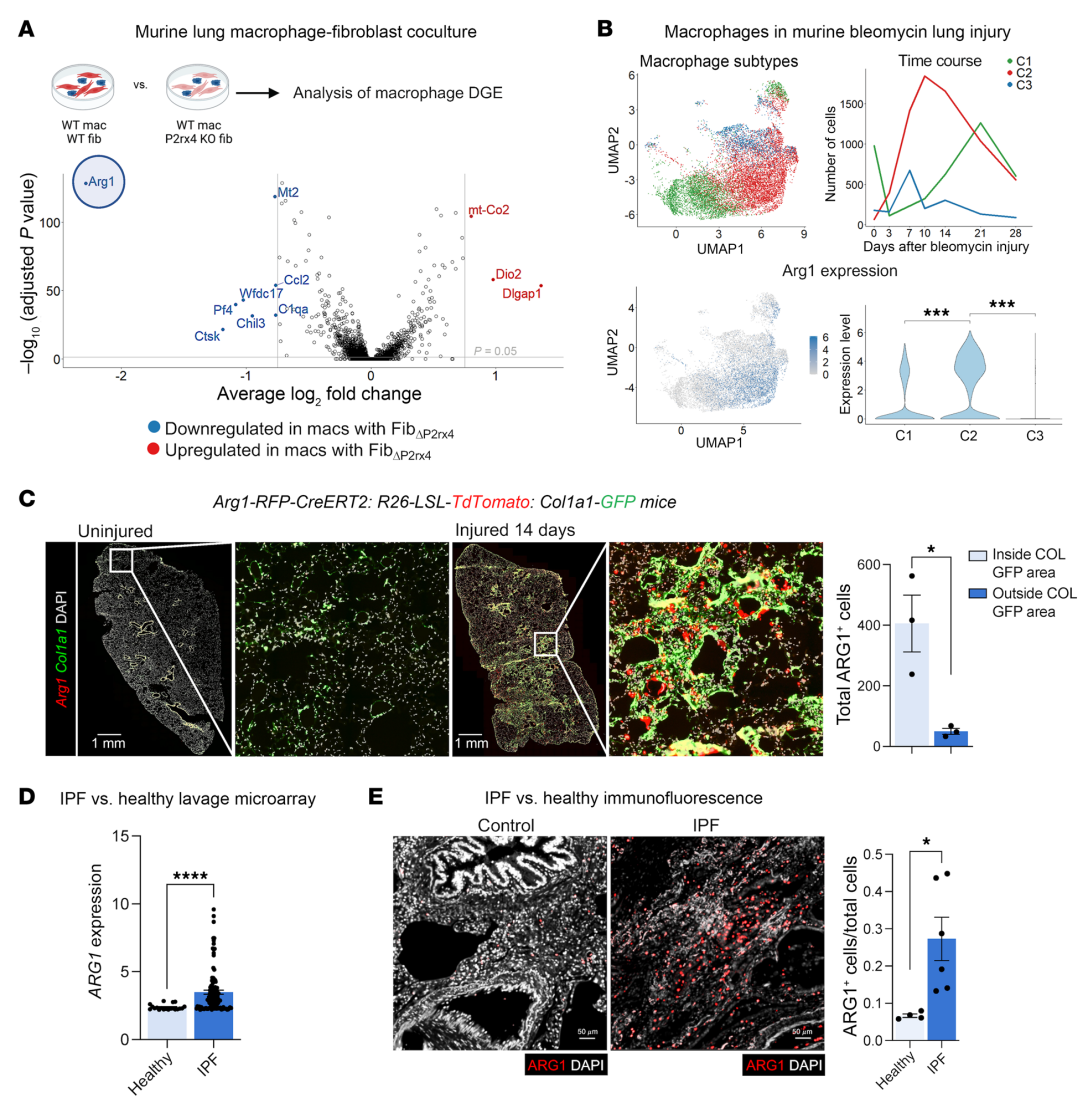
ARG1-expressing cells localize to the fibrotic niche in murine and human lung. (**A**) Volcano plot for macrophages (mac) from scRNA-Seq of macrophage-fibroblast cocultures for WT macrophages and fibroblasts (fibs) versus WT macrophages cocultured with *P2rx4* KO (*Pdgfrb-Cre P2rx4^fl/fl^*) fibroblasts. Significance was determined by Wilcoxon’s rank-sum test corrected for multiple comparisons by the Bonferroni method. Color-labeled genes had *P* < 0.05 and absolute value of log_2_(fold change) > 0.75. Data represent 2 separate cocultures. DGE, differential gene expression. (**B**) Analysis of macrophages from bleomycin lung injury (data reanalyzed from Strunz et al., ref. 6). Top left: Annotation of macrophages from multiple time points, according to C1, C2, or C3 macrophage annotation (as described by Aran et al., ref. 2): C1, alveolar macrophages; C2, transitional monocyte-derived macrophages; C3, monocyte-derived macrophages). Top right: Proportions of C1, C2, and C3 across time. Bottom left: Feature plot of *Arg1* expression. Bottom right: Violin plot of *Arg1* expression according to cluster (C1, C2, and C3). ***P < 0.001 by Student’s *t* test. (**C**) Immunofluorescence imaging of tamoxifen-induced *Arg1-RFP-CreERT2 LSL-tdTomato-Col1a1-GFP* mice at 14 days after bleomycin injury. Quantification shows total *Arg1*^+^ cells inside and outside contiguous *Col1a1*-*GFP*^+^ areas. *n* = 3 mice per condition. *P < 0.05 by Student’s *t* test. (**D**) *ARG1* expression by microarray analysis of gene expression of BAL cells from healthy patients (*n* = 20) and patients with IPF (*n* = 112) from GSE70867 (9). ****P < 0.0001 by Mann-Whitney test. (**E**) Representative immunofluorescence of human healthy control and IPF lung sections (*n* = 4 and 6, respectively) for ARG1. Scale bars: 50 μm. The quantification shows the proportion of ARG1^+^ cells (i.e., ARG1^+^/total DAPI count) for each sample. *P < 0.05 by Student’s *t* test. (**C**–**E**) Data are reported as mean ± SEM.

The most downregulated macrophage gene when fibroblast *P2rx4* was deleted was *Arg1*, a marker of alternatively activated macrophages (Figure 1A). Analysis of lung macrophages in a published scRNA-Seq time course after bleomycin lung injury (6) showed maximal *Arg1* in the “C2,” transitional macrophage compartment we previously demonstrated was localized to the fibrotic niche after injury (2) (Figure 1B). C2 cells expressed higher levels of *Arg1* and also the “Fab5” marker genes that were recently found to be associated with a core profibrotic macrophage program (7) (Supplemental Figure 1, C–E), and they were transcriptomically similar to recruited and interstitial macrophages (8) (Supplemental Figure 1F). Interestingly, the macrophages from our cocultures also expressed higher levels of interstitial macrophage than did alveolar macrophage genes (Supplemental Figure 1G). To determine whether *Arg1*^+^ cells localized to areas of fibrosis, we injured mice expressing reporter alleles for *Arg1* and for the fibroblast marker *Col1a1*. Confocal imaging revealed *Arg1*^+^ cells in proximity to clusters of activated fibroblasts in the fibrotic niche (Figure 1C).

To further profile *Arg1* expression in myeloid cell subsets in mice, we performed flow cytometry with *Arg1* reporter mice. This analysis confirmed that *Arg1* was expressed 10 days after bleomycin injury in macrophages expressing CD11B and CD64, markers consistent with monocyte-derived macrophages (Supplemental Figure 2, A–C). Canonical neutrophils expressing LY6G did not express *Arg1*, which we confirmed by immunofluorescence for the neutrophil marker S100A8 (Supplemental Figure 2D). However, we did note emergence of a small myeloid population of *Arg1*^+^ cells expressing LY6G that also expressed CD64 and CD11C, although this population was about 10-fold smaller in number than monocyte-derived macrophages (Supplemental Figure 2E). Taken together, these data confirm expression of *Arg1* in myeloid cells in the mouse lung fibrotic niche.

**Figure 2:**
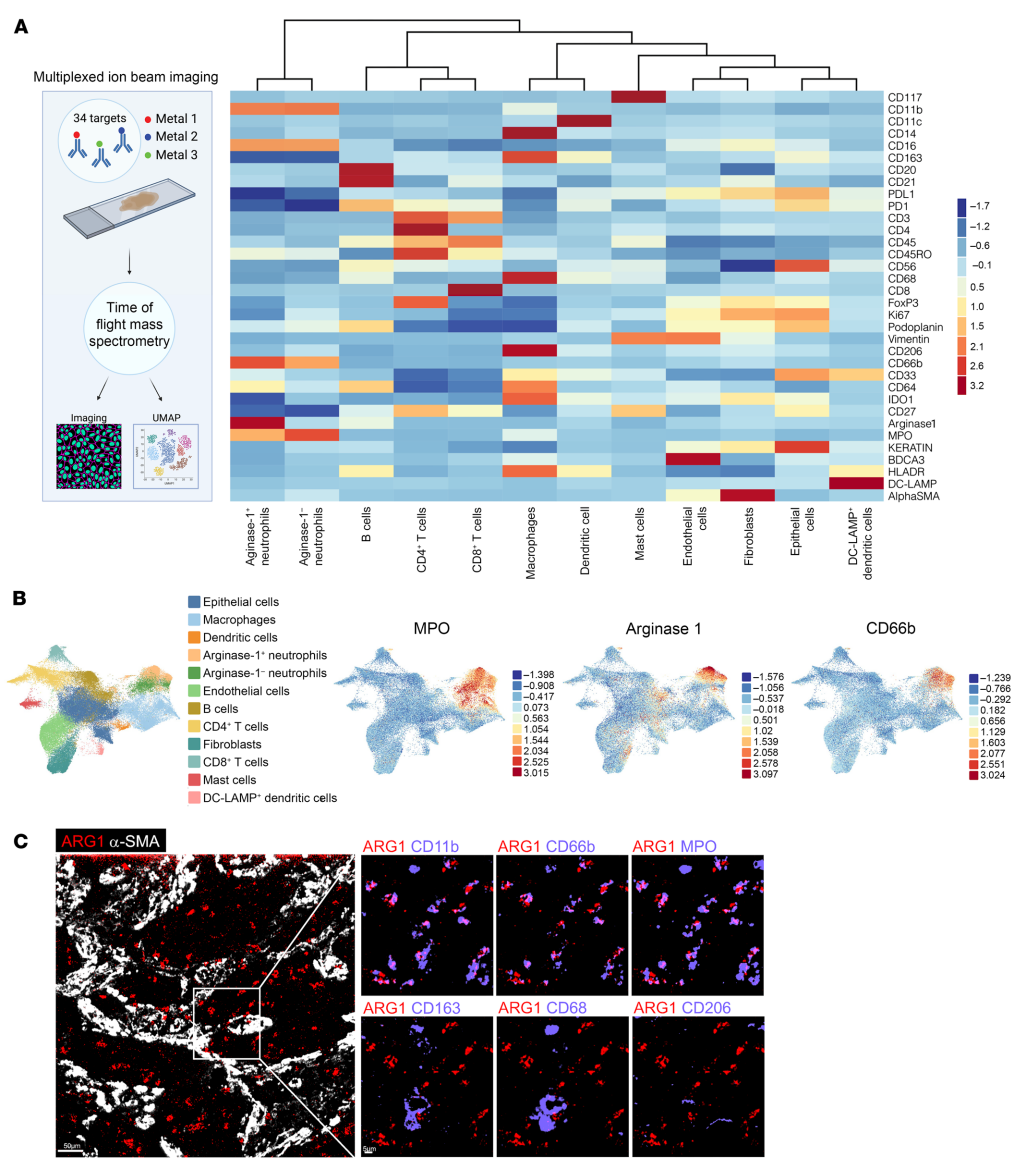
ARG1 is expressed predominantly by neutrophils in IPF. (**A**) Schematic of MIBI and a heatmap of scaled marker expression from MIBI of sections from 5 IPF explanted lungs. (**B**) MIBI UMAPs by cluster and individual markers. (**C**) MIBI images for individual markers within a representative FOV of IPF lung. Scale bars: 50 μm (left panel), 5 μm (right panels).

To determine the relevance of ARG1 to IPF, we first interrogated a published data set of BAL cell gene expression by microarray (9). This analysis confirmed higher *ARG1* expression in IPF compared with healthy control lungs (Figure 1D). We then found increased numbers of ARG1^+^ cells by immunofluorescence of IPF lung explants acquired at the time of clinical transplantation compared with deceased donor lungs not known to have lung disease (Figure 1E and Supplemental Table 1). In human lung, ARG1 has been found to be expressed in neutrophils, based on data from lung cancer specimens (10–12). However, deep phenotyping in IPF lung tissue of ARG1-expressing cell types has not been undertaken, to our knowledge. Therefore, to comprehensively profile the cell types expressing ARG1 in IPF, we performed multiplexed ion beam imaging (MIBI) (13–16), a technique in which isotopically pure elemental metal reporters are conjugated to antibodies and detected spatially in combination with mass spectrometry. We applied MIBI to sections from 5 IPF explanted lungs using anti-ARG1 antibody and 33 other antibodies, selecting markers primarily for the purpose of defining major immune cell types. The MIBI data showed that ARG1^+^ cells predominantly expressed the neutrophil-specific markers CD66B and MPO but not the macrophage markers CD68, CD206, and CD163 (Figure 2, A–C, and Supplemental Table 2). Importantly, neutrophils have been associated with increased mortality risk in individuals with IPF (17–20). However, their function in fibrogenesis has not been well studied.

### ARG1 regulates availability of ornithine, a profibrotic substrate.

**Figure 3:**
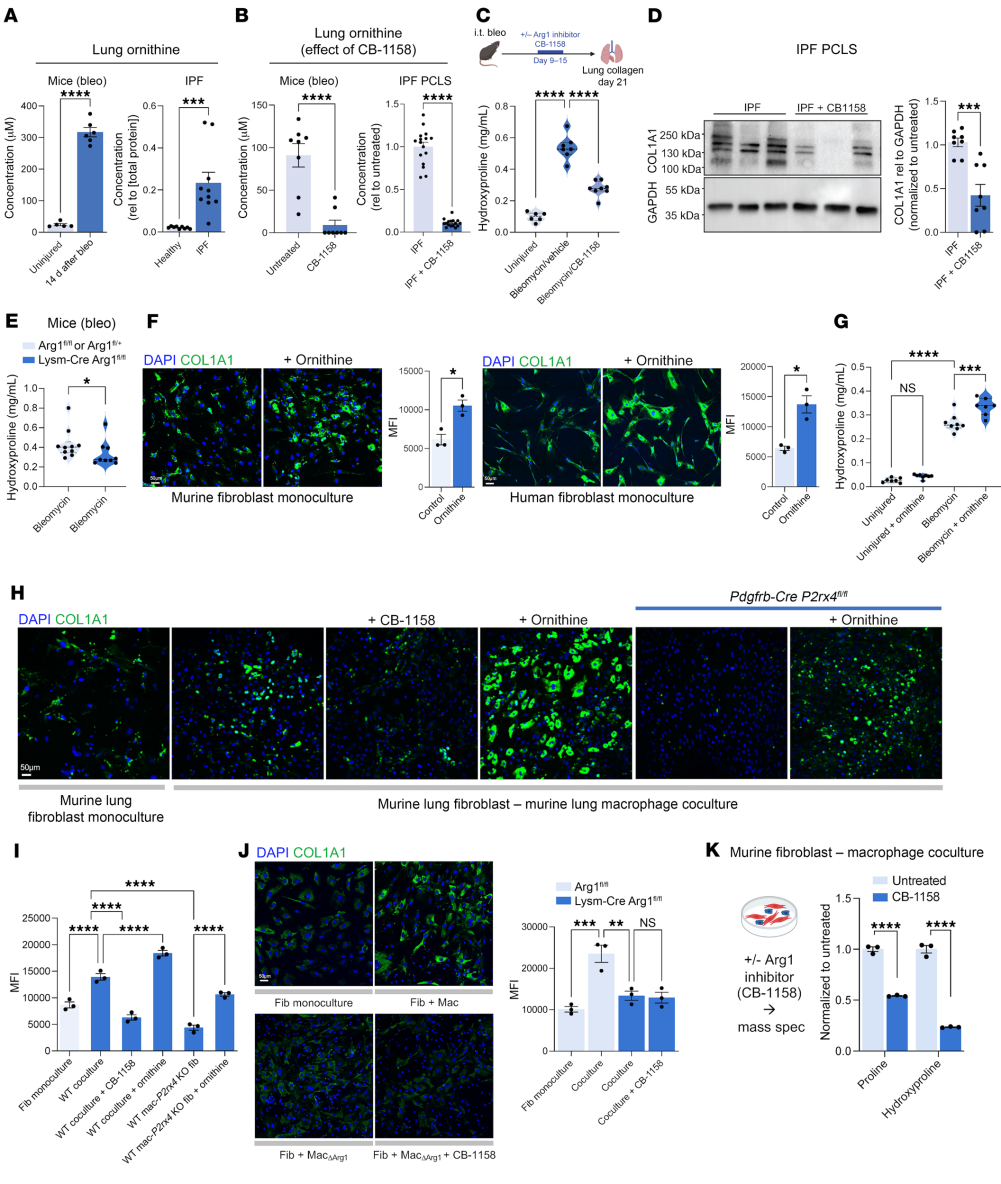
ARG1 regulates lung collagen via ornithine production. (**A**) Ornithine concentration measured in lysates of mouse lung (*n* = 5 uninjured and *n* = 6 injured mice), healthy human donor lung (*n* = 9), and lung explants of patients with IPF (*n* = 10). ***P < 0.001, ****P < 0.0001 by Student’s *t* test. (**B**) Ornithine concentration with and without CB-1158 treatment of lungs of bleomycin-injured mice (*n* = 8 in each condition) and cultured IPF PCLS (*n* = 16 slices from a total of 3 patients). ****P < 0.0001 by Student’s *t* test. (**C**) Hydroxyproline assay for collagen content of lungs from injured and uninjured WT mice treated with or without the ARG1 inhibitor CB-1158. Mice were dosed with 100 mg/kg CB-1158 twice daily during days 9–15 after bleomycin (bleo) administration. *n* = 6, 7, and 8 mice, respectively, left to right. ****P < 0.0001 by 1-way ANOVA with post hoc Šídák’s multiple-comparison test. Median, upper, and lower quartiles indicated by dashed lines. i.t., intratracheal. (**D**) Representative immunoblot for COL1A1 from lysates of precision-cut IPF lung slices cultured for 24 hours with or without ARG1 inhibitor CB-1158. Quantitation is for slices from a total of 3 patients. ***P < 0.001 by Student’s *t* test. (**E**) Hydroxyproline assay for collagen content of lungs from mice 21 days after bleomycin injury. *n* = 10 and 9 mice, respectively, left to right. *P < 0.05 by Mann-Whitney test. Median, upper, and lower quartiles indicated by dashed lines. (**F**) Left: COL1A1 immunofluorescence of monocultured mouse lung fibroblasts with or without ornithine treatment. Quantification is for 3 separate cultures each. *P < 0.05 by Student’s *t* test. Right: COL1A1 immunofluorescence of monocultured human lung fibroblasts with or without ornithine treatment. Quantification is for 3 separate cultures each. *P < 0.05 by Student’s *t* test. (**G**) Hydroxyproline assay for collagen content of lungs from injured WT mice treated with or without ornithine (2 g/kg) twice daily by ornithine gavage during days 7 through 20. *n* = 8 and 7 mice, respectively, left to right. ***P < 0.001, ****P < 0.0001 by 1-way ANOVA with post hoc Šídák’s multiple-comparison test. Median, upper, and lower quartiles indicated by dashed lines. (**H**) Representative samples of COL1A1 immunofluorescence of mouse lung macrophage-fibroblast cocultures from WT or fibroblast-specific *P2rx4*-KO (*Pdgfrb-Cre P2rx4^fl/fl^*) mice treated with or without CB-1158 or ornithine. (**I**) Quantitation of MFI from (**H**). *n* = 3 biological replicates per condition. ****P < 0.0001 by 1-way ANOVA with post hoc Šídák’s multiple-comparison test. (**J**) Representative samples of COL1A1 immunofluorescence of mouse lung macrophage-fibroblast cocultures treated with or without CB-1158. Mac, *Arg1^fl/fl^* control mice; Mac_ΔArg1_, macrophages isolated from *Lysm-Cre Arg1^fl/fl^* mice. Quantification is for 3 separate cultures each. **P < 0.01, ***P < 0.0001 by 1-way ANOVA with post hoc Šídák’s multiple-comparison test. (**K**) Relative quantities of proline and hydroxyproline in lysates of murine primary lung fibroblasts isolated after coculture with macrophages, with or without CB-1158 inhibitor treatment, quantified by liquid chromatography–mass spectrometry. *n* = 3 biological replicates for macrophages and fibroblasts, respectively. ****P < 0.0001 by 1-way ANOVA with post hoc Šídák’s multiple-comparison test. (**A**, **B**, **D**, **F**, **I**–**K**) Data are reported as mean ± SEM. Spec, spectrometry. Scale bars: 50 μm.

To understand ARG1’s function, we considered first that ornithine generated from arginine by ARG1 can serve as a substrate for the synthesis of proline (21), a major constituent of collagen. Remarkably, we found that ornithine was markedly increased both in mouse lung 14 days after bleomycin injury compared with steady state and in IPF lung lysates compared with healthy control lungs (Figure 3A).

To test the effects of ARG1 and ornithine in fibrosis, we first used the small-molecule ARG1 inhibitor CB-1158 (22). We confirmed the on-target effect of CB-1158 by measuring lung ornithine levels after treatment. In both bleomycin-injured mice and precision-cut lung slices (PCLS) from explanted IPF lungs, the inhibitor decreased ornithine levels (Figure 3B). Next, we treated bleomycin-injured mice with CB-1158 during the fibrotic period. Importantly, we found that lung fibrosis was markedly decreased and body weight recovery improved with ARG1 inhibition (Figure 3C and Supplemental Figure 2F). We then prepared PCLS from explanted IPF lungs and treated them with CB-1158 for 24 hours, followed by immunoblot for COL1A1 in the RIPA buffer–soluble fraction representing the more soluble, newly synthesized collagen (23). COL1A1 expression measured by immunoblot of lung slice lysates was decreased by CB-1158 treatment compared with untreated control lungs, indicating suppression of collagen synthesis with ARG1 inhibition (Figure 3D). Retention of cells expressing ARG1 and neutrophil markers MPO and CD66B was confirmed in PCLS by immunofluorescence (Supplemental Figure 3A).

Finally, taking a genetic approach with macrophage-specific *Arg1*-KO mice in which Cre-mediated deletion efficiency was approximately 55% (Supplemental Figure 3B), we found that lung fibrosis was decreased after injury in KO mice compared with control mice (Figure 3E). Taken together, these data indicate ARG1 determines the pathologic accumulation of collagen in lung fibrosis.

We then tested the direct effect of ornithine on collagen expression. First, we found, by immunofluorescence in monocultured fibroblasts from both mouse and human lung (Figure 3F), that exogenous ornithine increased fibroblast collagen expression. To test the profibrotic potential of ornithine in vivo, we treated mice with ornithine by oral gavage, either at steady state or during the fibrotic phase after bleomycin injury. Ornithine treatment increased lung collagen production in the setting of injury but not at steady state, consistent with increased cellular demand for collagen synthetic precursors including proline during fibrogenesis (Figure 3G). We then found that macrophage-fibroblast coculture increased collagen expression compared with fibroblast monoculture, an effect that could be blocked with CB-1158 or fibroblast *P2rx4* KO and enhanced with ornithine (Figure 3, H and I). Furthermore, ornithine could rescue the CB-1158–dependent decreased collagen expression in cocultures (Supplemental Figure 3C). Finally, fibroblast coculture with *Arg1*-KO macrophages was associated with decreased collagen expression compared with WT macrophages, an effect that was not augmented with CB-1158 (Figure 3J). Taken together, these results indicate a direct profibrotic effect of ornithine in vitro and in vivo.

Ornithine is converted by ornithine aminotransferase (OAT) to glutamate-5-semialdehyde, which spontaneously cyclizes to pyrroline-5-carboxylate and is then reduced by P5C reductases to proline. Notably, OAT inhibition decreased coculture-dependent augmentation of collagen expression (Supplemental Figure 3C). Interestingly, coculture also increased α-SMA expression, but this effect was not augmented by ornithine or suppressed by OAT inhibition (Supplemental Figure 3D). These data indicate a dependence of collagen synthesis on ARG1 function and ornithine metabolism.

Finally, we directly tested the hypothesis that ARG1-mediated ornithine production was necessary for increasing fibroblast proline content, using the approach of macrophage-fibroblast coculture followed by isolation of fibroblasts for liquid chromatography–mass spectrometry to detect proline. Consistent with our hypothesis, ARG1 inhibition with CB-1158 decreased fibroblast proline content in cocultures (Figure 3K). Taken together, these results suggest macrophage ARG1 drives fibrosis by producing ornithine, which serves as a substrate for fibroblast proline synthesis, augmenting collagen expression.

### ARG1 is regulated by IL-6.

**Figure 4:**
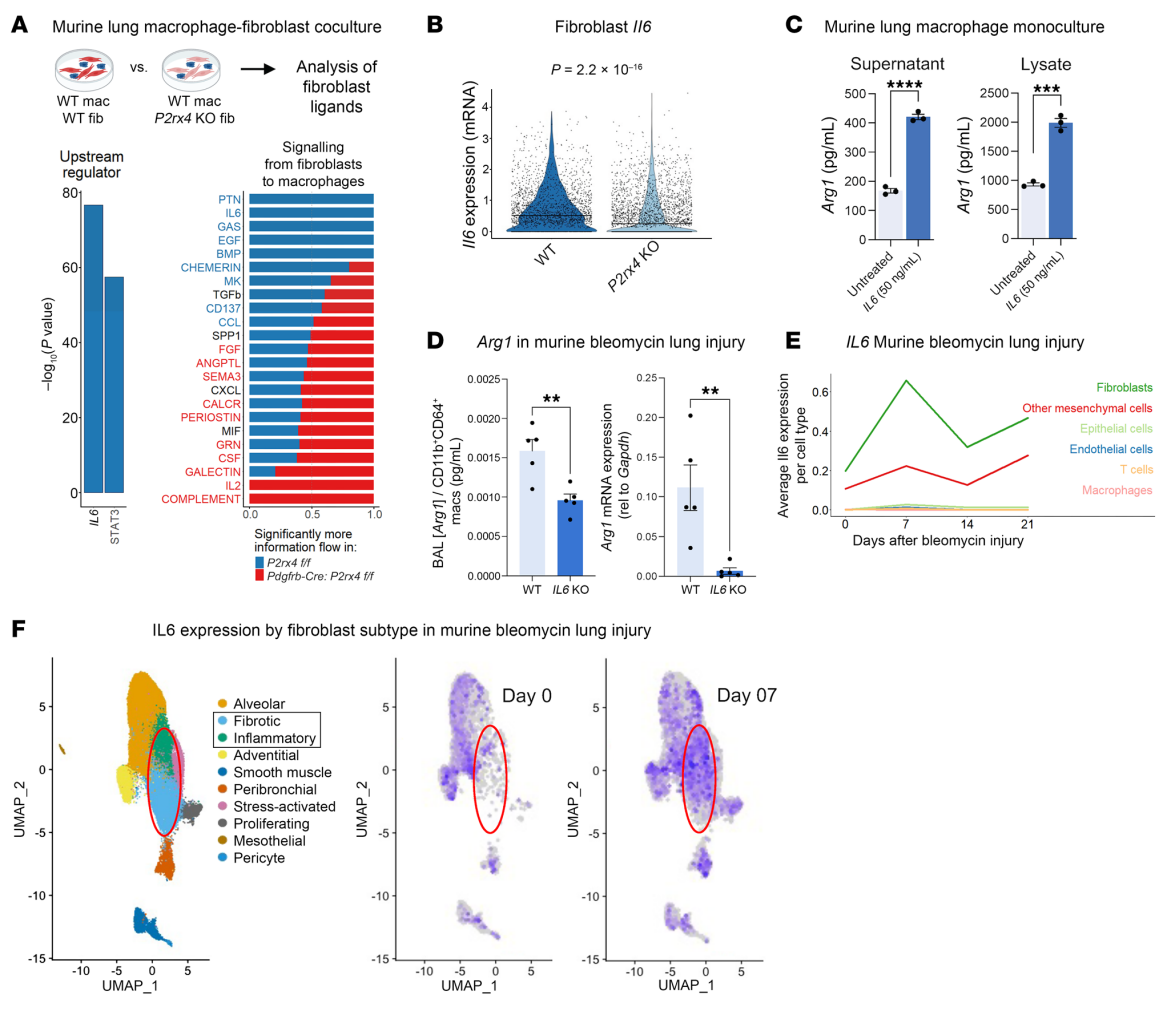
IL-6 is necessary for ARG1 expression after bleomycin injury in mice. (**A**) Left: Ingenuity Pathways Analysis of predicted upstream regulators for macrophages cultured with WT relative to *P2rx4*-KO fibroblasts (fib). Right: CellChat plot comparing WT and *P2rx4*-KO conditions. Significance was determined by Wilcoxon’s test, with *P* < 0.05 being statistically significant. Data represent 2 separate cocultures. (**B**) Violin plot for *Il6* expression in WT relative to *P2rx4*-KO fibroblasts from cocultures. Line shows median values. Data represent 2 separate cocultures. (**C**) ARG1 ELISA of cell lysates and conditioned media collected from mouse lung macrophage (mac) monoculture 72 hours after IL-6 treatment. *n* = 3 biological replicates per condition. ***P < 0.001, ****P < 0.0001 by Student’s *t* test. (**D**) Left: ARG1 ELISA of BAL fluid normalized to lung CD11B^+^CD64^+^ macrophage count taken at day 14 from bleomycin-injured WT or *Il6*-KO mice. Right: Arg1 qPCR of CD11B^+^CD64^+^ macrophages. *n* = 5 mice per condition. **P < 0.01 by Student’s *t* test. (**E**) Quantification of *Il6* expression by cell type in cells that underwent scRNA-Seq directly after isolation from the lung at steady state and multiple time points after injury: reanalysis of merged data from Strunz et al. (6) and Tsukui et al. (29) normalized by Sctransform (62). (**F**) UMAP of fibroblast subtypes (left) and *Il6* feature plots at steady state (center) and 7 days after bleomycin injury (right), from Tsukui et al. (29). (**C**, **D**, and **F**) Data are reported as mean ± SEM.

To determine how macrophage ARG1 expression is regulated by signaling within the fibrotic niche, we returned to our previously described coculture data (Figure 1A) indicating that macrophages cocultured with *P2rx4*-KO fibroblasts had decreased *Arg1* expression. CellChat (24) and Ingenuity Pathway Analysis revealed suppression of IL-6–based paracrine signaling in the *P2rx4*-KO coculture condition (Figure 4A). We also found that *P2rx4*-KO fibroblasts expressed less *Il6* mRNA compared with WT fibroblasts (Figure 4B).

IL-6 is an important profibrotic factor and is a therapeutic target in clinical lung fibrosis (25–28). We first confirmed an increase of ARG1 in cultured mouse lung macrophages treated with IL-6 (Figure 4C). In vivo, we noted that *Il6*-KO mice had decreased macrophage numbers in the lung, consistent with a chemotactic effect of IL-6 (Supplemental Figure 3E). Nonetheless, even accounting for this lower cell count, lung ARG1 levels were disproportionately decreased in *Il6*-KO mice, as indicated by concentrations of ARG1 in bronchoalveolar lavage (BAL), normalized to CD11B^+^CD64^+^ macrophage count and by quantitative PCR (qPCR) measurement of *Arg1* relative to *Gapdh* in flow-sorted CD11B^+^CD64^+^ macrophages (Figure 4D). Of note, *Il6*-KO mice had decreased lung fibrosis after bleomycin lung injury (Supplemental Figure 3F). We then analyzed scRNA-Seq time-course data for multiple lung cell types and found that *Il6* was, indeed, highly expressed in fibroblasts relative to other cell types (Figure 4E). Furthermore, *Il6* was notably expressed in the emergent, disease-associated inflammatory and fibrotic fibroblasts observed in recent time-course data (29) (Figure 4F).

**Figure 5:**
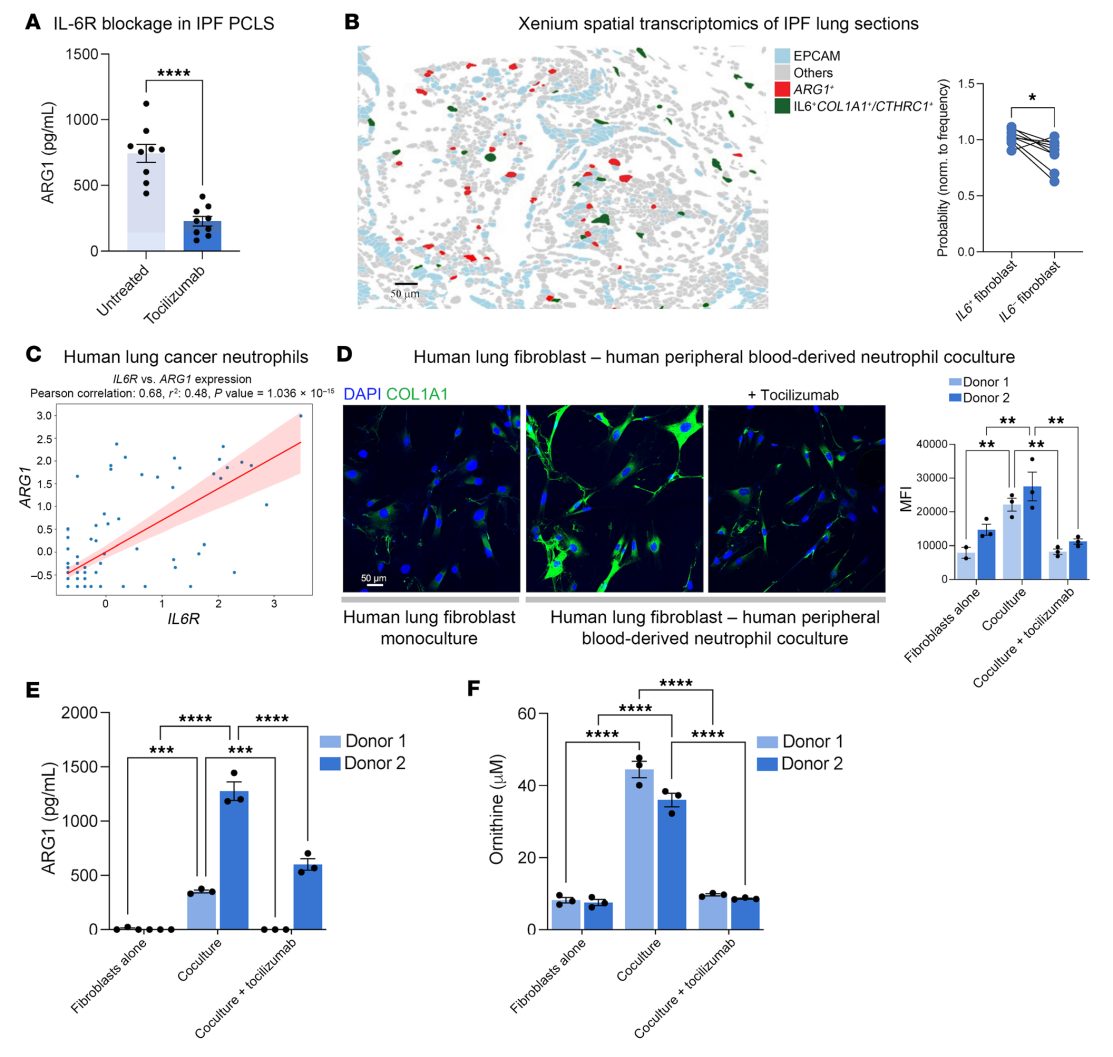
IL-6 is necessary for ARG1 expression in IPF lung and in cocultures of human blood–derived neutrophils and lung fibroblasts. (**A**) ARG1 ELISA of lysates of precision-cut IPF lung slices cultured for 24 hours with or without IL-6R–blocking antibody tocilizumab. Data represent 3 patients, with 3 slices per condition per patient assayed. ****P < 0.0001 by Student’s *t* test. (**B**) 10x Xenium spatial analysis: Left: Representative FOV showing cells expressing *ARG1* and *EPCAM*, and *IL6*^+^ fibroblasts (fibs) (defined by coexpression of either *COL1A1* or *CTHRC1*). Right: Proximity analysis of Xenium data. Data shown are for the probability ratio, P (exp|*ARG1*^+^)/P(exp), the probability of encountering an *IL6*^+^ fibroblast, normalized by fibroblast frequency, at the radial distance of 25 μm from *ARG1*^+^ cells. *P < 0.05 by paired Student’s *t* test. Data are for 9 separate patients. (**C**) Correlation scatter plot of tissue-resident neutrophils for *IL6R* and *ARG1* expression. The plot is a reanalysis of integrated scRNA-Seq data for NSCLC samples from 19 data sets from Salcher et al. (30). (**D**) COL1A1 immunofluorescence of human lung fibroblasts that were either monocultured or cocultured with human blood–derived neutrophils with or without IL-6R–blocking antibody (tocilizumab) treatment. Quantification is for 3 separate cultures each. Donor 1 and donor 2 represent separate donors for both neutrophils and fibroblasts. **P < 0.01 by 2-way ANOVA followed by Šídák’s multiple-comparison test. (**E**) ARG1 ELISA of conditioned media from the same cocultures as in **D**. ***P < 0.001, ****P < 0.0001 by 2-way ANOVA followed by Šídák’s multiple-comparison test. (**F**) Ornithine concentration of conditioned media from the same cocultures as in **D**. ****P < 0.0001 by 2-way ANOVA followed by Šídák’s multiple-comparison test. (**A**, **B**, **D**–**F**) Data are reported as mean ± SEM.

To test whether IL-6 could induce ARG1 in human lung, we first tested cultured PCLS from explanted IPF lungs. Treatment with the IL-6 receptor–blocking (IL-6R–blocking) antibody tocilizumab for 24 hours decreased ARG1 expression in IPF PCLS lysates (Figure 5A). To confirm that a paracrine interaction was spatially plausible, we performed spatial transcriptomic analysis of IPF samples and defined a proximity statistic to compare the probabilities of detecting *IL6*^+^ versus *IL6*^–^ fibroblasts in proximity to *ARG1*^+^ cells. Interestingly, at a plausible paracrine distance from ARG1^+^ cells (25 μm), fibroblasts were more likely than not to express *IL6* (Figure 5B).

We then interrogated published data sets to test whether *ARG1*^+^ lung neutrophils expressed *IL6R* and therefore could support IL-6 signaling. Extensive transcriptomic profiling of IPF neutrophils has not been reported to our knowledge; as an alternative, we analyzed data for non–small cell lung cancer (NSCLC) (*n* = 309 patients across 19 data sets) (30). This analysis revealed a strong correlation between *ARG1* and *IL6R* expression in neutrophils (Figure 5C).

Finally, we cocultured human peripheral blood–derived CD15^+^CD16^+^CD66B^+^CD14^–^ neutrophils (Supplemental Figure 3G) with human lung fibroblasts. After 24 hours of coculture, we removed the neutrophils, which are nonadherent cells, and measured collagen expression in the fibroblasts by immunofluorescence. Neutrophil coculture increased fibroblast collagen expression compared with fibroblast monoculture, and this increase could be blocked by IL-6R blockade with tocilizumab (Figure 5D). Notably, ARG1 and ornithine levels in the conditioned medium were increased by coculture compared with fibroblasts alone, and both were suppressed by tocilizumab (Figure 5, E and F). Taken together, these analyses are consistent with the hypothesis that fibroblast IL-6 induces neutrophil ARG1 expression in the human lung fibrotic niche.

### eATP signaling induces fibroblast IL-6.

**Figure 6:**
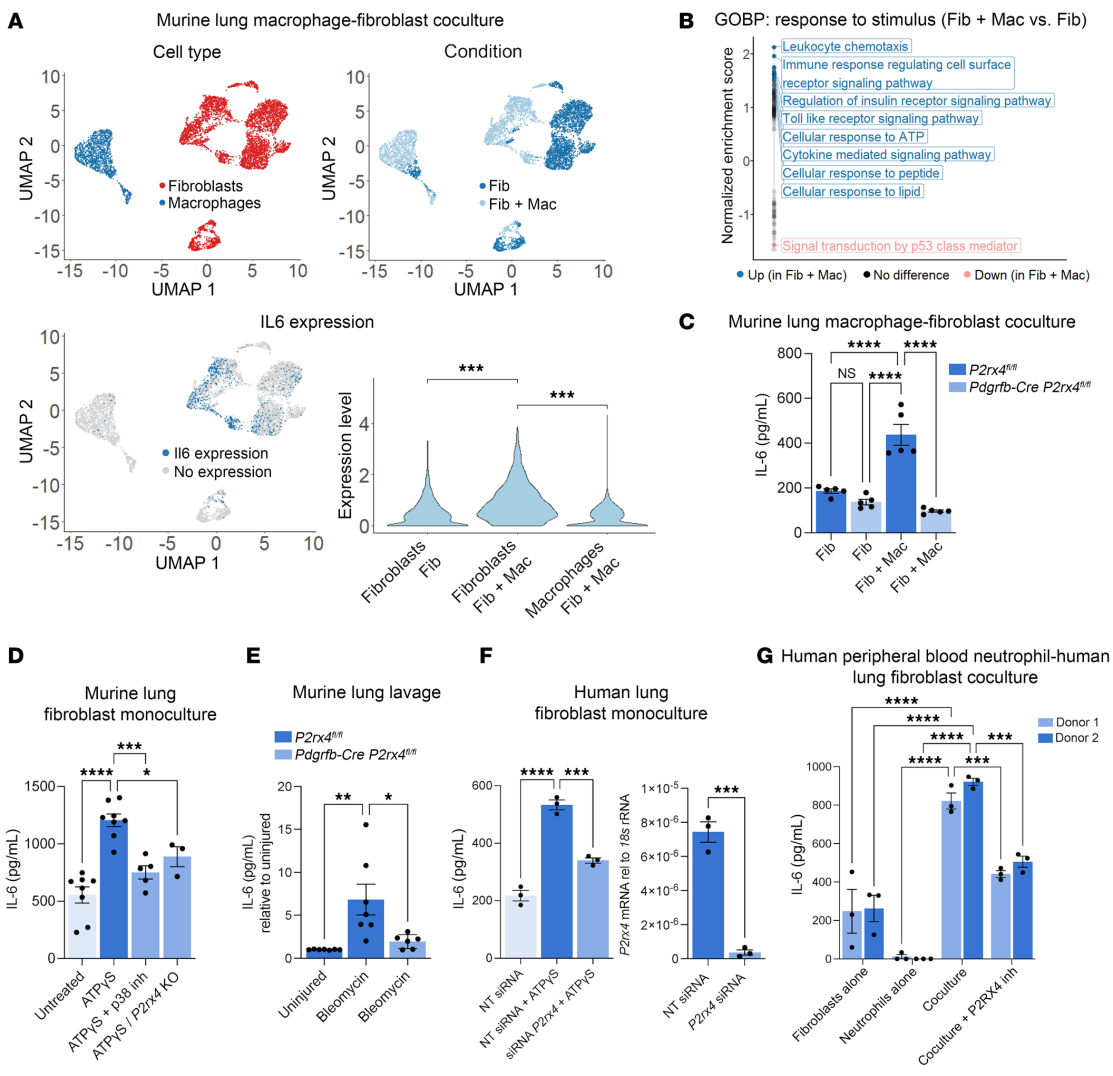
P2RX4 is necessary for IL-6 expression in mouse and human lung fibroblasts. (**A**) Top left: UMAP plot of scRNA-Seq for macrophage-fibroblast cocultures with SingleR-based cell type annotation (2) shown. Data represent 2 separate cultures for each condition. Top right: UMAP plot for data showing sample of origin. Fib, fibroblast monoculture; Fib + Mac, fibroblast coculture with macrophages. Bottom left: Feature plot showing *Il6* expression. Bottom right: Violin plot of *Il6* expression. ***P < 0.001 by Wilcoxon’s rank-sum test corrected for multiple comparisons by Bonferroni’s method. (**B**) Gene set enrichment analysis of fibroblast single cell transcriptomes in coculture with macrophages compared with fibroblast monoculture using Gene Ontology “Response to stimulus” pathways. (**C**) IL-6 ELISA of conditioned media from macrophage-fibroblast cocultures with or without fibroblast-specific *P2rx4* deletion. *n* = 5 biological replicates per condition. ****P < 0.0001 by 1-way ANOVA with post hoc Šídák’s multiple-comparison test. Data presented as mean ± SEM. (**D**) IL-6 ELISA of conditioned media from monocultured mouse lung fibroblasts from WT mice, with and without ATPγS and SB203580 (p38 MAP kinase inhibitor) treatment, or from fibroblast-specific *P2rx4*-KO (*Pdgfrb-Cre P2rx4^fl/fl^*) mice with ATPγS treatment. *n* = 8, 8, 5, and 3 biological replicates per respective condition. *P < 0.05, ***P < 0.001, ****P < 0.0001 by 1-way ANOVA with post hoc Šídák’s multiple-comparison test. (**E**) IL-6 ELISA of BAL from mice with or without fibroblast-specific *P2rx4* deletion. *n* = 7, 7, and 6 biological replicates, respectively, left to right. *P < 0.05, **P < 0.01 by 1-way ANOVA with post hoc Šídák’s multiple-comparison test. (**F**) Left: IL-6 ELISA of conditioned media from human donor lung fibroblast monocultures with *P2RX4* siRNA KD or nontargeting control siRNA (NT siRNA), with and without ATPγS treatment (left, *n* = 3 per condition). ***P < 0.001, ****P < 0.0001 by 1-way ANOVA with post hoc Šídák’s multiple-comparison test. Right: Quantification of KD by qPCR, *n* = 3 per condition. ***P < 0.001 by Student’s *t* test. (**G**) IL-6 ELISA of conditioned media from human donor lung fibroblast monocultures, human blood–derived neutrophil monocultures, or cocultures with or without P2RX4 inhibition with BAY-1797. Conditioned media were collected after 24 hours of culture. Each point represents a separate technical replicate. Neutrophils were derived from 2 separate blood donors, and fibroblasts were derived from 2 separate healthy lung donors. ***P < 0.001, ****P < 0.0001 by 2-way ANOVA followed by Šídák’s multiple-comparison test. Donor 1 and donor 2 represent separate donors for both neutrophils and fibroblasts. (**C**–**F**) Data are reported as mean ± SEM.

To test whether the eATP receptor P2RX4 regulates fibroblast IL-6 expression, we considered that myeloid cells including both macrophages and neutrophils can be a source of eATP (31). Thus, we performed scRNA-Seq of fibroblasts cocultured with macrophages or cultured alone. Interestingly, coculture increased fibroblast expression of *Il6* (Figure 6A), and the Gene Ontology pathway “Cellular Response to ATP” was enriched in fibroblasts in coculture compared with those in monoculture (Figure 6B). Importantly, IL-6 levels were higher in the conditioned medium of WT macrophages cocultured with WT versus *P2rx4*-KO fibroblasts (Figure 6C). Furthermore, direct treatment of cultured murine lung fibroblasts with ATPγS, a nonhydrolyzable form of ATP, increased IL-6 levels in the conditioned medium of WT but not *P2rx4*-KO lung fibroblasts, and this effect was blocked by inhibition of p38 MAP kinase, which mediates signaling downstream of P2RX4 (32, 33) (Figure 6D).

To evaluate these results in vivo, we tested mice with fibroblast-specific *P2rx4* KO after bleomycin injury. We found reduced IL-6 levels in the BAL fluid of *P2rx4*-KO compared with WT mice (Figure 6E). Furthermore, siRNA knockdown (KD) of *P2RX4* in human lung fibroblasts decreased IL-6 expression in response to ATPγS (Figure 6F). We also found that fibroblast *P2rx4* expression increased after bleomycin injury (Supplemental Figure 3H).

Finally, coculture of human peripheral blood–derived neutrophils with human lung fibroblasts increased IL-6 levels in the conditioned medium compared with fibroblasts or neutrophils alone. This effect could be inhibited by treatment with a small-molecule inhibitor of P2RX4 (BAY-1797) (34) (Figure 6G). Taken together, our findings indicate that, in both murine and human systems, eATP/P2RX4 signaling regulates fibroblast IL-6 expression, which, in turn, induces myeloid ARG1, resulting in ornithine loading of fibroblasts for proline synthesis and pathologic collagen expression.

## Discussion

In this study, we show that ARG1 and its enzymatic product ornithine have a profibrotic effect in the lung by driving synthesis of fibroblast proline, a key substrate for collagen expression. Imaging studies indicated that ARG1 is expressed more highly in IPF than in healthy human lung explants. KO and chemical inhibition of ARG1 demonstrated its profibrotic effect in murine lung injury, and these results were confirmed by chemical inhibition in IPF PCLS. Remarkably, we found that ornithine was increased in fibrotic lung tissue from mice and human patients with IPF. Furthermore, ornithine directly increased collagen expression in vivo and in cultured murine and human lung fibroblasts, with metabolic labeling studies confirming that ornithine was a substrate for proline synthesis. These data build on recent reports that ornithine was elevated in IPF plasma (35) and that the expression of ornithine aminotransferase, which converts ornithine to the proline precursor P5C, was correlated with areas of fibrosis in IPF lung (36).

Our findings also address how this profibrotic metabolism is initiated via paracrine crosstalk. In both murine and human fibrotic lung and in coculture systems, myeloid ARG1 expression was dependent on IL-6 expression in fibroblasts, which, in turn, was regulated by eATP/P2RX4 signaling. The recent literature has revealed a subset of fibroblasts expressing inflammatory genes in fibrosis, including cytokines (29, 37, 38). A pathologic function of fibroblast-derived cytokine expression is elucidated by our discovery that fibroblast IL-6 is necessary for ARG1 expression in myeloid cells, with the profibrotic consequence of ornithine loading of fibroblasts leading to increased collagen production.

The myeloid cell type expressing ARG1 varied by species. Using *Arg1* reporter mice, we found that *Arg1* was predominantly expressed in CD11B^+^CD64^+^ macrophages and not in canonical LY6G^+^ neutrophils. However, there was *Arg1* expression in a relatively smaller population of LY6G^+^CD64^+^CD11C^+^ cells, which may be an intermediate inflammatory myeloid lineage similar to a profile others have recently described in the setting of murine influenza infection (39). Future studies should more deeply profile these latter cells in comparison with ARG1^+^ cells from IPF, which expressed neutrophil markers, to determine whether they are phenotypically similar. Nonetheless, in both species, we confirmed a P2RX4/IL-6/ARG1, myeloid-mesenchymal circuit that is functionally important in lung fibrosis. Notably, blood and BAL neutrophilia has been associated with fibrosis progression and worse prognosis in IPF (17–20). Our work highlights ARG1 and ornithine metabolism as a neutrophil-dependent pathway that should be developed as a potential therapeutic target for IPF.

The role of type 2 inflammation in fibrosis deserves mention with respect to ARG1. In schistosomiasis-induced fibrosis models, ARG1 deletion led to an increase in type 2 inflammation and consequently increased fibrosis because of an accumulation of arginine, which supports CD4^+^ T cell proliferation (40). However, in the bleomycin-induced sterile injury model we have used, the onset of type 2 inflammation is later than the period when most collagen deposition has already occurred and, therefore, is less relevant to fibrogenesis (41). Furthermore, we note that inhibition of type 2 inflammation with dual IL-4 and IL-13 blockade did not show efficacy in an IPF clinical trial (42).

There are limitations that should be considered when interpreting our results. First, our metabolomic studies indicate conversion of ornithine to proline, a key substrate for collagen synthesis; however, we note that ornithine conversion to polyamines with profibrotic potential remains a possibility not yet addressed by our results. Second, ARG2 is another arginase that could also contribute to ornithine loading of fibroblasts, and studies should focus on how both arginases are regulated across the temporal phases of fibrosis. Third, our findings are agnostic with respect to the site of ornithine generation: we detected ARG1 both in both the lysates and conditioned medium of IL-6–treated macrophages, which is consistent with the possibility of either intracellular ornithine production and export or extracellular production, per published reports (43, 44). Finally, regarding sources of eATP, we concede it is likely that multiple sources of eATP exist in the fibrotic niche, such as dying or dysfunctional cells, in addition to myeloid cells themselves. Our results, nonetheless, reveal that the DAMP signal eATP triggers expression of fibroblast IL-6, which induces profibrotic ARG1 in neighboring myeloid cells.

In conclusion, we show the dependence of lung fibrosis on ornithine deriving from myeloid ARG1 in mouse models and in functional studies of IPF lung. The importance of understanding the contribution of amino acid metabolism to fibrosis has been recognized in recent years (45), and our findings highlight the role of its immune regulation in the synthesis of pathologic collagen. These studies increase enthusiasm for targeting ARG1 as a therapeutic approach in IPF.

## Methods

### Sex as a biological variable.

For preclinical in vivo studies, we used both female and male mice, and experimental groups were balanced with respect to sex. Human lung samples were acquired from both male and female patients.

### Human lung tissues.

IPF lung samples were obtained as explants at the time of lung transplantation. Deceased-donor control lungs not known to have lung disease were made available by Donor Network West. Demographic data with respect to ethnicity and race (Supplemental Table 1) were derived from the electronic medical record and classified per NIH notice NOT-OD-15-089.

### Mice.

*P2rx4^fl/fl^* mice were previously generated by one of the study authors (RM) (46). *Arg1^fl/fl^* (47), *Lysm-Cre* (48), *Il6* KO (49), C57BL/6 WT, and *R26-LSLS-TdTomato* mice were obtained from The Jackson Laboratory. *Col1a1-GFP* mice were obtained from David Brenner (Sanford Burnham Prebys, La Jolla, California, USA) (50); *Pdgfrb-Cre* mice were obtained from Ralf Adams (University of Münster, Münster, Germany) (51), and *Arg1-RFP-CreERT2* (52) and *Arg1-YFP* (53) mice were obtained from Richard Locksley (UCSF, San Francisco, California, USA). All mice were on a C57BL/6 background and were maintained in a specific pathogen–free animal barrier facility at UCSF. All experiments were performed on 6- to 8-week-old, sex-matched mice.

### Mouse lung injury.

For lung injury, mice anesthetized with isoflurane were instilled intratracheally with bleomycin (Fresenius; 3 U/kg). In the case of ARG1 inhibition, bleomycin-injured mice were treated daily from day 9 to day 15 after injury with 100 mg/kg CB-1158 (Numidargistat dihydrochloride, HY-101979A; MedChemExpress) dissolved in water, by oral gavage. In the case of ornithine treatment, mice were treated twice daily by gavage with ornithine 2 g/kg dissolved in 100 mL of water.

### Murine macrophage-fibroblast coculture.

We prepared cocultures of macrophages and fibroblasts that were isolated from the lungs of mice 7 days after bleomycin lung injury. To make single-cell suspensions, minced lung tissue was resuspended in RPMI medium with 0.2% collagenase (10103586001, Roche), 2000 U/mL DNase I (4716728001, Roche), and 0.1 mg/mL Dispase II (4942078001, Sigma-Aldrich) for 1 hour at 37°C and then passed through a 70 μm filter (130-110-916, MACS SmartStrainers; Miltenyi Biotec), followed by 2 washes with PBS (10010023, Gibco). Macrophages were isolated by positive selection with 20 μL CD11B microbeads (130-049-601, Miltenyi Biotec) per 1 × 10^7^ cells, using LS MACS columns (130-042-401, Miltenyi Biotec). Isolated macrophages were cultured in DMEM with 10% FBS, 1% penicillin-streptomycin (Gibco), and 20 ng/mL M-CSF (315-02, Peprotech) for 2 days. Primary mouse lung fibroblasts were isolated by antibody-based negative selection of epithelial cells (biotin anti-mouse CD326 Epcam, clone G8.8; 118204, BioLegend), endothelial cells (biotin anti-mouse CD31, clone 390; 102404, BioLegend), immune cells (biotin anti-mouse CD45, clone 30-F11; 103104, BioLegend), pericytes and smooth muscle cells (biotin anti-mouse CD146, clone ME-9F1; 134716, BioLegend), and red blood cells (biotin anti-mouse Ter119, clone TER-119; 116204, BioLegend) with biotinylated antibodies and Dynabeads (MyOne Streptavidin T1; 65601, Thermo Fisher Scientific), as previously described (37). Fibroblasts were added to macrophages and cocultured at a 1:1 ratio in complete DMEM for 5 more days.

### Human lung fibroblast isolation.

Deceased-donor human lung tissue not used for transplant was minced in HBSS buffer supplemented with 0.2% collagenase (10103586001, Roche), 2,000 U/mL DNase I (4716728001, Roche), 0.1 mg/mL Dispase II (4942078001, Sigma-Aldrich), and 1% penicillin-streptomycin for 1.5 hours at 37°C and 5% CO_2_. Amphotericin B 1× (15290026, Gibco) was added to the dissociation solution for the last 30 minutes. Digested lung tissue was lysed further with a gentleMACS Dissociator (Miltenyi Biotec) using gentleMACS C tubes (130-093-237, Miltenyi Biotec) at the mLUNG-01 setting. The suspension was then passed through a 70 μm filter to obtain single cells. Cells were resuspended in PBS with 0.5% BSA and 2 mM EDTA.

For the negative selection of fibroblasts, the following antibodies were used: epithelial cells (biotin anti-human Epcam, clone 9C4; 324216, BioLegend), endothelial cells (biotin anti-human CD31, clone WM-59; 13-0319-82, Invitrogen), immune cells (biotin anti-human CD45, clone 2D1; 368534, BioLegend), and pericytes and smooth muscle cells (biotin anti-human CD146, clone P1H12; 361036, BioLegend). Fibroblasts were cultured in DMEM supplemented with 10% serum, 1% penicillin-streptomycin, and 1% amphotericin B.

### Human neutrophil isolation and coculture with fibroblasts.

Whole blood (10 mL) from heathy donors was collected in BD Vacutainer K2 EDTA Tubes (Vitalant) and used for primary neutrophil isolation, as previously described (54). Briefly, 7 mL of blood was layered on top of 7 mL of PolymorphPrep (AXS-1114683, Cosmo Bio USA) and centrifuged at 500*g* for 35 minutes at room temperature (RT) without braking. The peripheral blood mononuclear cells and plasma layers were aspirated and the neutrophil layer was collected. The cells were washed with PBS and centrifuged at 400*g* for 5 minutes. The cell pellet was resuspended in 3 mL of ACK lysis buffer (A1049201, Thermo Fisher Scientific) and centrifuged at 400*g* for 5 minutes. Neutrophil purity was 95%, confirmed by flow cytometry (Supplemental Figure 3F). The following antibodies were used: CD15 clone W6D3 (323039, BioLegend); CD66B clone G10F5 (B221034, BioLegend); CD16 clone 3G8 (560474, BD); and CD14 clone 63D3 (367118, BioLegend). The cells were finally resuspended in RPMI medium supplemented with 10% FBS and counted. Neutrophils were then added to human lung fibroblasts for 24 hours of coculture in RPMI medium with 10% FBS, with or without P2RX4 inhibitor BAY-1797 (1 μm; 7573, Tocris Bioscience) or IL-6R–blocking antibody tocilizumab (100 ng/mL; HY-P9917, MedChemExpress). The conditioned medium was retained for analysis after separation of the cellular fraction by centrifugation, and fibroblasts were fixed and permeabilized for immunofluorescence analysis.

### Flow cytometry and sorting.

Cell cocultures were trypsinized with 0.25% trypsin and resuspended in 1× PBS with 0.5% BSA and 2mM EDTA. Single-cell suspensions were prestained with Fc blocker for 10 minutes in ice and then were stained at 1:300 with anti-CD64 (PE anti-mouse CD64, clone X54-5/7.1; 139304, BioLegend) and anti-PDGFRA (PE-Cy7, anti-mouse CD140a, clone APA5; 25-1401-82, Invitrogen) antibodies for 40 minutes. DAPI was used to distinguish dead cells.

For mouse lung cell suspensions, whole-lung single-cell suspensions were prepared by harvesting lung lobes into 5 mL of HBSS with 40 μL of Liberase (0.1 U/mL; 5401127001, Roche) and 20 μL of DNase 1 (10 mg/mL; 10104159001, Roche), followed by gentleMacs automated tissue dissociation and digestion for 30 minutes at 37°C on a shaker. Digested samples were processed on the gentleMacs using the “lung2” program, passed through 70 μm filters, and washed, followed by red blood cell lysis and final suspension in FACS buffer. Cells were counted using a NucleoCounter (ChemoMetic). All samples were stained in 96-well V-bottom plates.

Single-cell samples were first incubated with antibodies to surface antigens for 30 minutes at 4°C in a 50 μL staining volume. Flow cytometry was performed on a BD LSRFortessa X-20 flow cytometer. Fluorochrome compensation was performed with single-stained UltraComp eBeads (01-2222-42, Invitrogen). Samples were forward scatter area/side scatter area gated to exclude debris, followed by forward scatter height/forward scatter area gating to select single cells and Draq7 viability dye (BioLegend) to exclude dead cells. Monocyte-derived macrophages were identified as CD19^–^, LY6G^–^, NK1.1^–^, Siglec-F^–^, CD11B^+^, and CD64^+^. ARG1^+^ cells were identified by the presence of eYFP. Data were analyzed using FlowJo software (TreeStar) and compiled using Prism (GraphPad Software). The following monoclonal antibodies were used: anti-CD45 (30-F11, BioLegend); anti-CD11B (M1/70, BioLegend); anti-CD11C (N418, BioLegend); anti-NK1.1 (PK136, BioLegend); anti-CD19 (6D5, BioLegend); anti-CD64 (X54-5/7, eBiosciences); anti-LY6G (1A8, BioLegend); anti-Siglec-F (E50-2440, BD Biosciences); anti–I-A/I-E (MHCII) (M5/114.15.2, BioLegend); and anti-CCR2 (475301, BD).

In the case of quantifying and sorting CD45^+^CD11B^+^CD64^+^ monocyte-derived macrophages in bleomycin-injured WT and *Il6*-KO mouse lung cell suspensions, 50,000 microbeads (Invitrogen/Thermo Fisher Scientific) were added to each sample for quantification of absolute live CD45^+^CD11b^+^CD64^+^ cells. Lavage cell pellets were added to the suspensions prior to sorting, and cells were sorted on a BD FACSAria II cell sorter.

### Quantitative real-time PCR analysis.

Human lung fibroblasts were lysed in 300 μL of TRIzol reagent (10296010, Ambion Life Technologies) to obtained RNA. The RNA (600 ng per sample) was used to prepare cDNA, using iScript Reverse Transcriptase Supermix (1708841, Bio-Rad Laboratories). qPCR was performed for the target genes using SYBR Green Super Mix (4309155, Applied Biosystems). The following primers were used: *P2RX4* forward: GAGATTCCAGATGCGACCACT, *P2RX4* reverse: ACCCGTTGAAAGCTACGCAC; and *18S* rRNA forward: GTAACCCGTTGAACCCCATT, *18S* rRNA reverse: CCATCCAATCGGTAGTAGCG. For sorted CD45^+^CD11b^+^CD64^+^ murine lung monocyte-derived macrophages, the following primers were used: *Arg1* forward: CTCCAAGCCAAAGTCCTTAGAG, *Arg1* reverse: AGGAGCTGTCATTAGGGACATC; and *Gapdh* forward: AGTATGACTCCACTCACGGCAA, *Gapdh* reverse: TCTCGCTCCTGGAAGATGGT.

### siRNA KD.

Healthy human fibroblasts were treated with 5 μM siRNA (ON-TARGET plus Human P2RX4 SMARTpool; L-006285-00-0010, Dharmacon) or control (ON-TARGET plus nontargeting siRNA; D-001810-01-05, Dharmacon) using DharmaFECT in serum-free medium for 12 hours, followed by supplementing with DMEM with 10% serum. Cells were incubated for an additional 48 hours. Treated cells were harvested for either RNA isolated or stimulated with 100 μM ATPγS for 12 hours, and conditioned medium was collected for human IL-6 ELISA assay.

### IL-6 ELISA.

BAL fluid from mice or conditioned medium from cultured murine or human cells was collected for measurement of IL-6 with a Duoset ELISA kit (DY406-05, R&D Systems) for mouse and the Duoset ELISA kit (R&D Systems, DT206-05) for human, following the manufacturer’s protocol.

### Arginase 1 ELISA.

Arg1 levels were measured from conditioned medium of cultured macrophages or from BAL, using a Mouse Arginase 1 ELISA Kit (Abcam, ab269541) following the manufacturer’s protocol. For human tissue, ARG1 levels were measured from RIPA buffer lysates of IPF PCLS or conditioned medium from cells cultured for 24 hours, with or without tocilizumab (100 ng/mL; HY-P9917, MedChemExpress), using the Human Arginase 1 ELISA Kit (BMS2216, Thermo Fisher Scientific) following the manufacturer’s protocol.

### Histology and immunofluorescence imaging.

Mouse lungs were inflated with a solution consisting of 30% sucrose solution mixed 1:1 with OCT compound (4585, Thermo Fisher Scientific), fixed in 4% formaldehyde at 4°C for 4 hours, washed in PBS, and then submerged in 30% sucrose solution overnight at 4°C. Next, the tissue was incubated in the 1:1 solution of 30% sucrose and OCT overnight, followed by changing to OCT for 2 hours, and then a tissue block was frozen after embedding in OCT. Sections (5 μm) were cut from OCT-embedded tissue. In some cases, frozen sections were incubated with S100A8 antibody (AF3059, R&D Systems) in staining buffer (1% BSA, 0.5% Triton X-100 in PBS), washed, and then incubated with anti-rabbit secondary antibody (Invitrogen). Slides were washed with PBS and mounted on antifade DAPI mounting medium. Human lung sections (5 μm thick) from patient FFPE blocks were collected for IHC staining. Slides were stained using Opal Manual IHC kit (PerkinElmer). After deparaffinization, antigen retrieval was performed in AR buffer for 45 seconds at 100% power followed by 15 minutes at 20% power. After blocking, slides were incubated with primary antibodies, ARG1 (93668, Cell Signaling Technology), MPO (88757, Cell Signaling Technology), and CD66B (EPR25354, Abcam) overnight at 4°C. Polymer HRP was introduced to slides for 10 minutes, followed by signal amplification using Opal570 (NEL810001KT, Akoya Biosciences) for 10 minutes at RT. The slides were then counterstained with DAPI mounting medium and scanned using with ×10 and ×40 objectives of a Leica inverted widefield microscope.

### Cultured cells.

Macrophages and fibroblasts or fibroblasts alone, isolated from mouse lung as detailed earlier in Methods, were cultured on glass coverslips and, in some cases, treated with CB-1158 (1 μM), ornithine (1 mM), or 1 μM OAT inhibitor 5-FMOrn dihydrochloride (HY154021A, MedChemExpress). Cells were fixed with 4% paraformaldehyde, permeabilized with 1% BSA and 0.5 % Triton X-100 in PBS, and incubated with primary antibodies (COL1A1, 720265, Cell Signaling Technology; α-SMA, A5228, Sigma-Aldrich) followed by a wash, secondary antibodies, another wash, and mounting. Imaging was performed on a Leica SP8 laser scanning confocal microscope. Fluorescence signal was quantified with Imaris software for cellular areas.

### PCLS preparation and Western blot.

PCLS were obtained from human IPF tissue sections. Briefly, IPF tissue was injected with 2% low-melting agarose (50111, Lonza) and then submerged in ice-cold PBS to allow solidification of agarose. Lung slices (400 μm) were generated using a Compresstome device (VF-310-OZ, Precisionary Instruments). Slices were kept in complete DMEM for 2 hours to allow the release of agarose, followed by changing to fresh complete DMEM with 10% FBS and 1% penicillin-streptomycin. Lung slices were treated with 50 μM CB-1158 for 24 hours. After incubation, slices were minced using a tissue homogenizer. Cells were lysed in Pierce RIPA buffer (89901, Thermo Fisher Scientific) with a protease inhibitor cocktail (1861278, Thermo Fisher Scientific). Protein (10 μg) was run on 10% SDS-PAGE (4561034, Bio-Rad) and transferred to a PVDF membrane (88520, Thermo Fisher Scientific). The membrane was incubated with COL1A1 antibody (720265, Cell Signaling Technology) overnight at 4°C. Blots were washed in 1× TBST and incubated with peroxidase-conjugated goat anti-rabbit (1:20,000; AS28177, Anaspec) for 4 hours at 4°C. Blots were developed using the SuperSignal West Pico Chemiluminescent substrate (34080, Thermo Fisher Scientific) in ChemiDoc XRS+ gel imaging system (Bio-Rad). Quantification of bands was done using ImageJ.

### Hydroxyproline assay.

Mice were euthanized and lungs were excised and snap-frozen. Isolated lung samples were homogenized and incubated with 50% trichloroacetic acid (T6399, Sigma-Aldrich) on ice for 20 minutes. Samples were then incubated in 12N HCL (A144, Thermo Fisher Scientific) overnight at 110°C. Dried pellets were reconstituted in distilled water with constant shaking for 2 hours at RT. Samples were then mixed with 1.4% Chloramine T (85739, Sigma-Aldrich) and 0.5 M sodium acetate (241245, Sigma-Aldrich) in 10% 2-propanol (A416, Thermo Fisher Scientific) and incubated with Ehrlich’s solution (03891, Sigma-Aldrich) for 15 minutes at 65°C. Absorbance was quantified at 550 nm, and concentration was calculated using a standard curve of commercial hydroxyproline (H5534, Sigma-Aldrich).

### scRNA-Seq library preparation and sequencing.

The PIPseq T2 3′ Single Cell RNA Kit (version 3.0, cultured cells; version 4.0, directly sequenced cells) was used for pretemplated instant partitioning (PIP) to capture single-cell mRNA transcripts with PIP beads according to manufacturer’s protocol (FB0001026, Fluent Biosciences). After 5 days of coculture, single-cell suspension of cells was obtained by trypsinization, followed by washing in 1× PBS. Cells were washed in ice-cold PIPseq Cell Suspension Buffer (FB0002440, Fluent Biosciences). Cells were counted and stained with Trypan blue to confirm >90% viability. Single-cell library preparation was performed using the manufacturer-recommended default protocol and settings. The sequencing libraries were submitted to the UCSF Center for Advanced Technology (NovaSeq X; Illumina) or Novogene (NovaSeq 6000; Illumina) for sequencing. The demultiplexed FASTQ files were aligned to mouse genome (mm10) using PIPseeker 1.0.0 (Fluent Biosciences).

After sequence alignment, we observed approximately 50% of input cells being called when PIPseeker sensitivity level was set to 3, which was near the inflection point of the barcode rank plot. The original FASTQ files, the quality reports, and the expression matrix outputs of PIPseeker have been deposited in Gene Expression Omnibus (GEO).

### scRNA-Seq data analysis.

The Seurat-Guided Clustering Tutorial (March 27, 2023) was followed to convert our expression matrixes into Seurat objects (Seurat, version 4.3.0) (55). For quality control, we removed the cells with fewer than 200 genes or more than 10,000 genes and larger than 5% mitochondrial content. Seurat objects were integrated following Seurat’s Introduction to scRNA-Seq Integration (March 27, 2023), selecting the top 2,000 variable features as integration anchors. Cell doublets were removed with the package DoubletFinder (2.0.3) (56). Following the Seurat-Guided Clustering Tutorial (March 27, 2023), we selected the top 2,000 highly variable genes to obtain the cell uniform manifold approximation and projection (UMAP) coordinates and group the cells into clusters with a sensitivity of 0.5 (55). Cell types were annotated using the package SingleR 1.10.04 (2) using the ImmGen database (57) as a reference. The Gene Set Enrichment Analysis was performed using the package enrichR 3.1 (58) (FDR < 0.05), with Gene Ontology data taken from the database “GO_Biological_Process_2021” (59). Cell communication pathways analysis was performed using the package CellChat (1.6.1) (24). Upstream regulator prediction was done using the Ingenuity Pathway Analysis software (QIAGEN) (60). Differentially expressed genes for macrophages with *P* less than 0.05 and average log_2_(fold change) greater than 0.75 were used for analysis.

### MIBI.

To prepare slides, serial 5-μm FFPE sections were cut onto 1 glass and 1 gold slide. Both slides were baked at 70°C overnight and deparaffinized in 3 washes of fresh xylene and rehydrated in ethanol (EtOH) (twice in 100% EtOH, twice in 90% EtOH, once in 80% EtOH, and once in 70% EtOH) and distilled water (twice) washes. Washes were performed in a Leica ST4020 Linear Stainer. An antigen retrieval slide chamber was prepared by diluting 10× Tris with EDTA antigen retrieval buffer 1:10 in deionized water (diH_2_O). The prepared slide chamber was added to a PT module filled with PBS and preheated to 75°C. The rehydrated slides were run in the preheated PT module at 97°C for 40 minutes, then cooled to 65°C in the PT module. The prepared slide chamber was then removed from the PT module and cooled at RT for 30 minutes. Slides were washed twice in 1× TBS-Tween.

### Glass-slide IHC.

Tissues were encircled by PAP pen boarders and blocked with 5% donkey serum diluted in TBS IHC wash buffer for 1 hour at RT in a moisture chamber. Wash buffer was aspirated from the slide, and ARG1 primary antibody (715001, Ionpath) was stained overnight at 4°C overnight in the moisture chamber. The following day, primary antibody was aspirated, and slides were washed twice with 1× TBS-T for 5 minutes, blocked with 3% peroxide buffer for 15 minutes at RT, and washed again twice with 1× TBS-T for 5 minutes each wash before incubation with anti-rabbit secondary antibody for 1 hour at RT. Slides were washed twice with 1× TBS-T for 5 minutes before visualization with 100 μL 3,3′-diaminobenzidine (DAB) for 5 minutes at RT. The DAB reaction was stopped by tapping waste into a contained waste bin and then washing the slide into a slide chamber filled with diH_2_O three times for 30 seconds each wash. The slide was then stained with hematoxylin for 1 minute at RT and washed with tap water twice for 30 seconds each time. The slide was then dehydrated by washing in EtOH (once in 70% EtOH, once in 80% EtOH, twice in 95% EtOH, and twice in 100% EtOH), and xylene (twice) before coverslipping.

### Gold slide staining.

Gold slides were transferred to the Sequenza Immunostaining Center Staining System (Electron Microscopy Sciences). Endogenous biotin-binding proteins with blocked with avidin/biotin-blocking reagents for 30 minutes at RT. We added 5% donkey serum to the top of the chamber to wash out the avidin-blocking reagents and block additional nonspecific antibody binding sites for 1 hour at RT. Primary and secondary antibodies panels were assembled with appropriate volumes of each titrated antibody and a final concentration of 0.05 M EDTA. The complete cocktails were filtered through a prewet 0.1 μm Ultrafree MC Spin Filter (Sigma-Aldrich), and then the primary antibody cocktail (Supplemental Table 2) was added to the Sequenza top chamber and incubated overnight at 4°C. The secondary antibody cocktail was stored at 4°C. The following day, the slides were washed by adding 1× TBS-T to the Sequenza chamber twice before adding the secondary antibody cocktail to the Sequenza chamber for 1 hour at RT. Gold slides were removed from the Sequenza chamber and washed three times with 1× TBS-T for 5 minutes each wash, once with filtered 2% glutaraldehyde for 5 minutes, three times with filtered 1× Tris pH 8.5, twice with filtered diH_2_O, once with 70% EtOH, once with 80% EtOH, twice with 90% EtOH, and twice with 100% EtOH. Slides were allowed to dry at RT for 10 minutes before being stored in a vacuum chamber before undergoing MIBIscope analysis.

### Image acquisition and processing.

Gold slides were loaded into the MIBIscope (Ionpath) and fields of view (FOVs) were selected by matching tissue topography to regions of interest with ARG1^+^ staining from the serial IHC glass slide. FOVs were acquired at fine resolution, with a dwell time of 1 second at a resolution of 0.39 μm per pixel. Image quality control was performed by following the Angelo Lab toffy pipeline (https://github.com/angelolab/toffy). Analysis was performed by following the Angelo Lab ark pipeline (https://github.com/angelolab/ark-analysis).

### Spatial transcriptomics (10x Xenium).

FFPE-preserved sections of lung tissue were prepared for spatial transcriptomics imaging on the Xenium platform by following 10x Genomics protocols CG000582 Rev E and CG000584 Rev A. Briefly, protocol CG000582 was followed to prepare the slides for the imaging run by first hybridizing the probe panel of choice. Here, the standard human lung panel available from 10x Genomics (catalog 1000601) was supplemented with a custom panel specific for lung disease states, including pulmonary fibrosis (Supplemental Table 3). After probe hybridization, annealed probes were ligated together to create circular fragments, and the circularized probes were amplified with a rolling circle PCR. After rolling circle amplification, slides were DAPI stained for nuclei and then placed in the Xenium Analyzer with run reagents, following protocol CG000584. The gene panel was uploaded to the Xenium Analyzer and a primary image was taken of the slides. The Xenium analyzer processed imaging data during the run, identified cell boundaries, and generated image files along with transcript by location matrices for further downstream analysis by the Seurat 10x Xenium protocol.

### Proximity analysis of ARG1^+^ cells and IL-6^+^ fibroblasts.

The output files of the Xenium analyzer were converted to an AnnData object that contained the cell centroid coordinates and raw transcript counts. A filter of 10 counts/cell and 5 cells/gene was applied to filter out low-quality cells and sparsely detected genes. Counts were normalized to 1 × 10^4^ and log-transformed. Cells were then grouped into 1 of the following 3 categories: (a) *ARG1*^+^; (b) *IL-6*^+^*ARG1*^–^ and either *CTHRC1*^+^ or *COL1A1*^+^; or (c) *IL-6*^+^*ARG1*^–^ and *CTHRC1*^+^ or *COL1A1*^+^, with positive expression of a marker defined as having nonzero counts.

### Co-occurrence probability ratio.

The Analyze Xenium data tutorial in Squidpy (version 1.5.0) (61) was followed to compute the co-occurrence probability ratio of ARG1^+^ and fibroblasts. We performed 50% random subsampling of the data 5 times to compute the statistics and generate CIs bounded by the minimum and maximum probability ratio generated by the subsamples. The co-occurrence probability ratio was computed at 25 μm between cells. Using the implementation in the 1.5.0 release of Squidpy, for each radial distance, the ratio of cells belonging to category exp (exp being any of the 3 aforementioned cell categories) out of all cells within the given distance of an *ARG1*^+^ cell was averaged across all *ARG1*^+^ cells to compute the conditional probability in the numerator P(exp | *ARG1*^+^), and the denominator P(exp) was computed similarly, but averaging probabilities were computed by centering around every cell in the sample. Previous releases of Squidpy implemented a version of this test in the function gr.co_occurrence that used discrete interval bins (only including cells within 2 consecutive choices of radial distances). We chose to use inclusive intervals (including all cells within a given radial distance) for a more robust calculation of the co-occurrence probability ratio. We implemented our method into the codebase of Squidpy, and it is now the default implementation of the function gr.co_occurrence, starting in release 1.5.0.

### Analysis of published data sets.

For the bleomycin time course, the time series of single-cell data after bleomycin lung injury were obtained from Tsukui et al. (29) and Strunz et al. (6). These samples were processed and merged with Seurat (version 4.3.0) using the function SCTransform to correct for batch effects and was annotated with SingleR 1.10.044 using the ImmGen database as a reference to identify cell types. For macrophage annotation in vivo, we annotated macrophages from Strunz et al. (6) according to the macrophage subtypes (C1, C2, and C3) defined by Aran et al. (2). and by Li et al. (8), using SingleR (2). For the BAL microarray, microarray RNA data from bronchoalveolar cells of healthy individuals and patients with IPF were extracted from the GPL14550 data set within the GSE70867 repository (9). The differentially expressed genes between the IPF and healthy samples were obtained by following the R workflow provided by the NCBI GEO2R platform using GEOquery (2.66.0) and limma (3.54.2) packages.

### Gene expression of cultured mouse-lung-derived macrophages.

The top markers of macrophage subtypes as defined by Li et al. (8) and Aran et al. (2) were quantified in our CD11B^+^, CSF1-treated macrophages (from the WT-WT coculture condition).

### Analysis of lung tumor neutrophils.

A high-resolution single-cell atlas of tumor-associated neutrophils in NSCLC was obtained from Salcher et al. (30). The scANVI algorithm–based integrated NSCLC transcriptome atlas provided more than 1.2 million cells from 19 studies and 309 patients. The metadata embedded with Leiden-clustering and cell-type annotations were used to identify and subset neutrophils. The subset data were log transformed and scaled, followed by filtering for cells expressing at least 1 of the following genes: *ARG1* and *IL6R*. The Pearson correlation coefficient, *P* value, and *R*^2^ value were calculated for the correlation between *ARG1* and *IL6R*.

### Ornithine measurement.

Cultured PCLS from IPF explanted lungs lysed in water, IPF lung samples lysed in RIPA buffer, or mouse-lung homogenized and lysed in RIPA buffer were used to measure ornithine, following manufacturer’s protocol (IS I-1000R, Immusmol).

### Liquid chromatography–mass spectrometry metabolomics.

Primary murine lung macrophages and fibroblasts were cocultured with or without 1 μM CB-1158. Extracts of isolated fibroblasts were used to calculate protein equivalents by resuspension in 0.2 M NaOH, heated to 95°C for 25 minutes, and determined via BCA (23225, Pierce). Dried metabolites were resuspended in 50% acetonitrile (ACN)/water, and 1/10th of the volume was loaded onto a Luna 3 μm NH2 100A (150 × 2.0 mm) column (Phenomenex). The chromatographic separation was performed on a Vanquish Flex (Thermo Scientific) with mobile phases A (5 mM NH_4_AcO pH 9.9) and B (ACN) and a flow rate of 200 μL/min. A linear gradient from 15% A to 95% A over 18 minutes was followed by 9 minutes of isocratic flow at 95% A and reequilibration to 15% A. Metabolites were detected with a Thermo Scientific Q Exactive mass spectrometer run with polarity switching (+3.5 kV/–3.5 kV) in full scan mode with an mass-to-charge ratio range of 65 to 975. TraceFinder 4.1 (Thermo Fisher Scientific) was used to quantify the targeted metabolites by area under the curve using expected retention time and accurate mass measurements (<5 ppm). Values were normalized to cell number and sample protein concentration.

### Statistics.

One-way ANOVA followed by post hoc Šídák’s multiple-comparison test was used for comparisons among more than 2 groups, and 2-tailed Student’s *t* test was used for comparison between 2 groups. A *P* value of less than 0.05 was considered significant. Analysis appropriately corrects for multiple comparisons and repeated measures.

### Study approval.

Experiments in mice were performed in accordance with approved protocols by the UCSF Institutional Animal Care and Use Committee. The studies with human tissues described in this article were conducted according to the principles of the Declaration of Helsinki. Written, informed consent was obtained from all study participants, and the studies were approved by the UCSF IRB. With respect to deceased-donor control explanted lungs, because samples were acquired from deceased individuals, the study is not considered human participants research per UCSF and NIH policy.

### Data availability.

The newly generated single-cell sequencing data are available at GSE242510. Values for all data points in graphs are reported in the Supporting Data Values file.

## Author contributions

PY performed or assisted in all the experiments and in figure preparation. JGO performed single-cell sequencing experiments and analysis and figure preparation. KCC and SP performed single-cell library preparation and sequencing, supervised by ARA. NB and AB assisted in functional experiments with lung tissues and cells. PD assisted in human neutrophil isolation and coculture experiments under the supervision of SKP. XY assisted PY in coculture experiments and microscopy under the supervision of BL. JN performed flow cytometry of mouse lung cells, supervised by ABM. KB, TJ, and JTW performed mouse breeding and development of crosses for experiments. TT and DS assisted in analysis of single-cell sequencing data from mouse lung fibroblasts. MM and HT provided deceased-donor control lung tissues, supervised by MAM. LM, AG, CJM, and VCA performed Xenium spatial transcriptomic analysis, supervised by WLE. CC and AW designed and implemented spatial proximity analysis of Xenium data. RM generated and provided *P2rx4^fl/fl^* mice. WT and SJT performed MIBI analysis under the guidance of TGB. PJW provided lung explants from patients with IPF and helped interpret associated imaging data. KMT designed and performed metabolomic assays, assisted by PY, and contributed to the conceptualization of the ornithine mechanism. MB conceived of the work, supervised experimental planning and execution, and wrote the manuscript with input from PJW and KMT.

## Funding support

- US Department of Defense (grant W81XWH2110417 to MB).
- UCSF Bakar Aging Research Institute (investigator grant to MB).
- Longevity Impetus Grants from Norn Group (to MB and JGO).
- UCSF Bakar Aging Research Institute investigator award (to MB).
- The Nina Ireland Program for Lung Health Innovative Grant Program (to PY).
- NIH (grants 1R01HL142701-01 to ABM and R01AI172754 to SKP).

## Supplementary Material

Supplemental data

Unedited blot and gel images

Supplemental tables 1-3

Supporting data values

## Version 1 08/28/2025

In-Press Preview

## Version 2 11/03/2025

Electronic publication
