# Supplementary material for: Myeloid-mesenchymal crosstalk drives ARG1-dependent profibrotic metabolism via ornithine in lung fibrosis
Source: J Clin Invest. 2025 Aug 28;135(21):e188734. doi: 10.1172/JCI188734 (PMC12578403; doi:10.1172/JCI188734)
Supplement: Supplemental data [file jci-135-188734-s096.pdf]

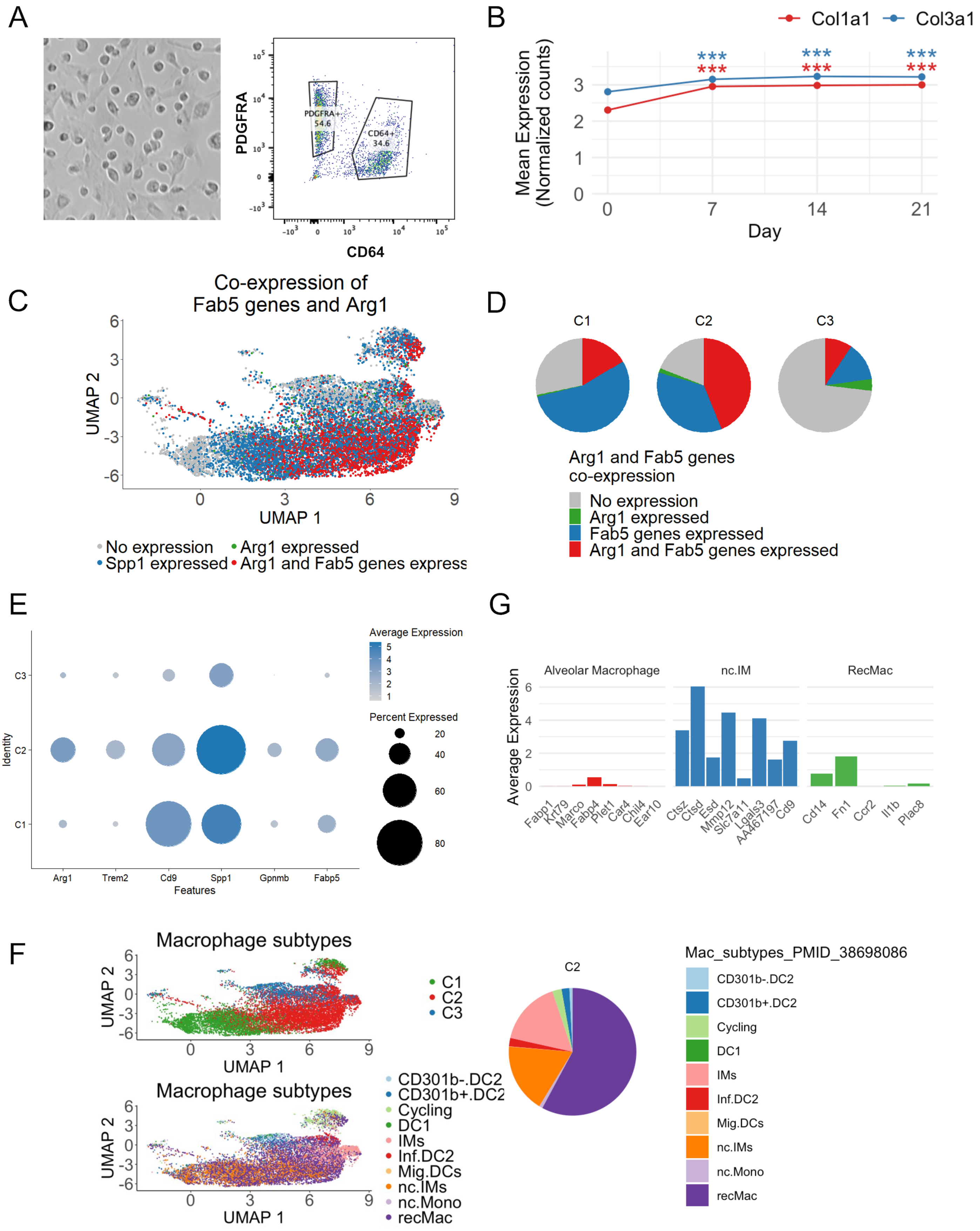
A
PDGFRA
CD64
PDGFRA+ 54.6
CD64+ 34.6
B
Col1a1
Col3a1
Mean Expression (Normalized counts)
Day
***
C
Co-expression of Fab5 genes and Arg1
UMAP 2
UMAP 1
No expression
Arg1 expressed
Spp1 expressed
Arg1 and Fab5 genes express
D
C1
C2
C3
Arg1 and Fab5 genes co-expression
No expression
Arg1 expressed
Fab5 genes expressed
Arg1 and Fab5 genes expressed
E
Average Expression
Percent Expressed
Identity
Features
Arg1
Trem2
Cd9
Spp1
Gpnmb
Fabp5
G
Alveolar Macrophage
nc.IM
RecMac
Average Expression
Fabp1
Krt79
Marco
Fabp4
Plet1
Car4
Chil4
Ear10
Ctsz
Ctsd
Esd
Mmp12
Slc7a11
Lgals3
AA467197
Cd9
Cd14
Fn1
Ccr2
Il1b
Plac8
F
Macrophage subtypes
UMAP 2
UMAP 1
C1
C2
C3
CD301b-.DC2
CD301b+.DC2
Cycling
DC1
IMs
Inf.DC2
Mig.DCs
nc.IMs
nc.Mono
recMac
Mac_subtypes_PMID_38698086

Supplemental Figure 1

A) Left: Bright field image of cocultured primary macrophages and fibroblasts. Right: Flow cytometry for PDGFRA and CD64 with coculture cells.
B) Expression of collagen genes across time after bleomycin injury in lung fibroblasts from Tsukui et al. (1) ***$p<0.001$.
C) Feature plot of lung macrophages from Strunz et al. (2) with *Arg1* and *Spp1* expression as well as Fab5 profibrotic macrophage genes from Fabre et al. (3) indicated.
D) Pie charts show proportions C1, C2, or C3 lung macrophages in (C). C1=alveolar macrophages; C2=transitional monocyte-derived macrophages; C3=monocyte derived macrophages; from Aran et al. (4).
E) Dot plot showing lung macrophage profibrotic genes from Fabre et al. (3) in macrophages from Strunz et al. (2) annotated as either C1, C2, or C3 macrophages
F) Annotation of cells from (C) by transcriptomic comparison using SingleR(4) to interstitial macrophage subtypes form Li et al. (2).
G) Analysis of expression of macrophage marker genes in the scRNAseq data for our CD11B+, CSF1-treated cultured macrophages (from the WT-WT coculture condition) to determine their likeness to macrophage subtypes defined by marker genes for interstitial macrophages from Li et al. (5) (RecMac and nc.IM), or for alveolar macrophages from Aran et al. (4).

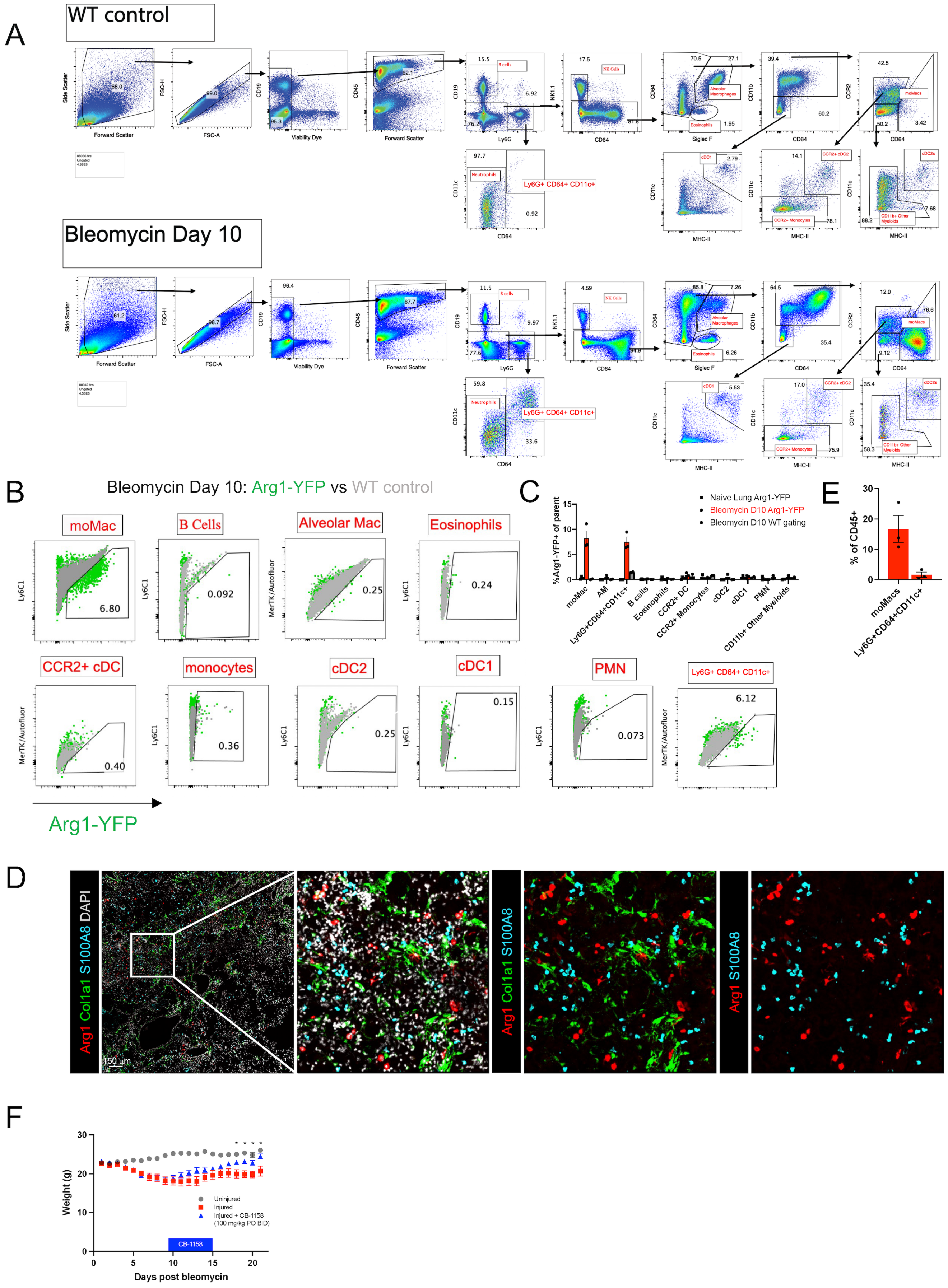
A
WT control
Bleomycin Day 10
B
Bleomycin Day 10: Arg1-YFP vs WT control
moMac
B Cells
Alveolar Mac
Eosinophils
CCR2+ cDC
monocytes
cDC2
cDC1
PMN
Ly6G+ CD64+ CD11c+
Arg1-YFP
C
Naive Lung Arg1-YFP
Bleomycin D10 Arg1-YFP
Bleomycin D10 WT gating
% Arg1-YFP+ of parent
E
% of CD45+
D
Arg1 Col1a1 S100A8 DAPI
Arg1 Col1a1 S100A8
Arg1 S100A8
150 μm
F
Weight (g)
Uninjured
Injured
Injured + CB-1158 (100 mg/kg PO BID)
CB-1158
Days post bleomycin

Supplemental Figure 2

A) Gating strategy for flow cytometry of lung cells at steady state. AM=alveolar macrophage (CD64+SiglecF+). MoMac=monocyte-derived macrophage (CD64+CD11B+SiglecF-).
B) Representative flow cytometry of mouse lungs from *Arg1-YFP* reporter and WT mice at steady state and 10 days post-bleomycin.
C) Plot of percentage of cells expressing YFP in each lineage, from flow cytometry corresponding to representative gating shown in (A) and (B). N=3 mice per condition. +/- SEM.
D) Lung immunofluorescence for neutrophil marker S100A8 from *Arg1-RFP-CreERT2: R26-LSL-TdTomato : Col1a1-GFP mice*, with no overlap between S100A8 and Arg1 detected. Data is representative of N=3 mice.
E) Plot of percentage of CD45+ cells accounted for by moMacs and LY6G+CD64+CD11C+ cells at day 10 post-bleomycin, from flow cytometry corresponding to representative gating shown in (B). N=3 mice per condition. +/- SEM.
F) Weight measured daily in CB-1158-treated mice (corresponding to Figure 3a). *$p<0.05$ by Student's t-test. +/- SEM.

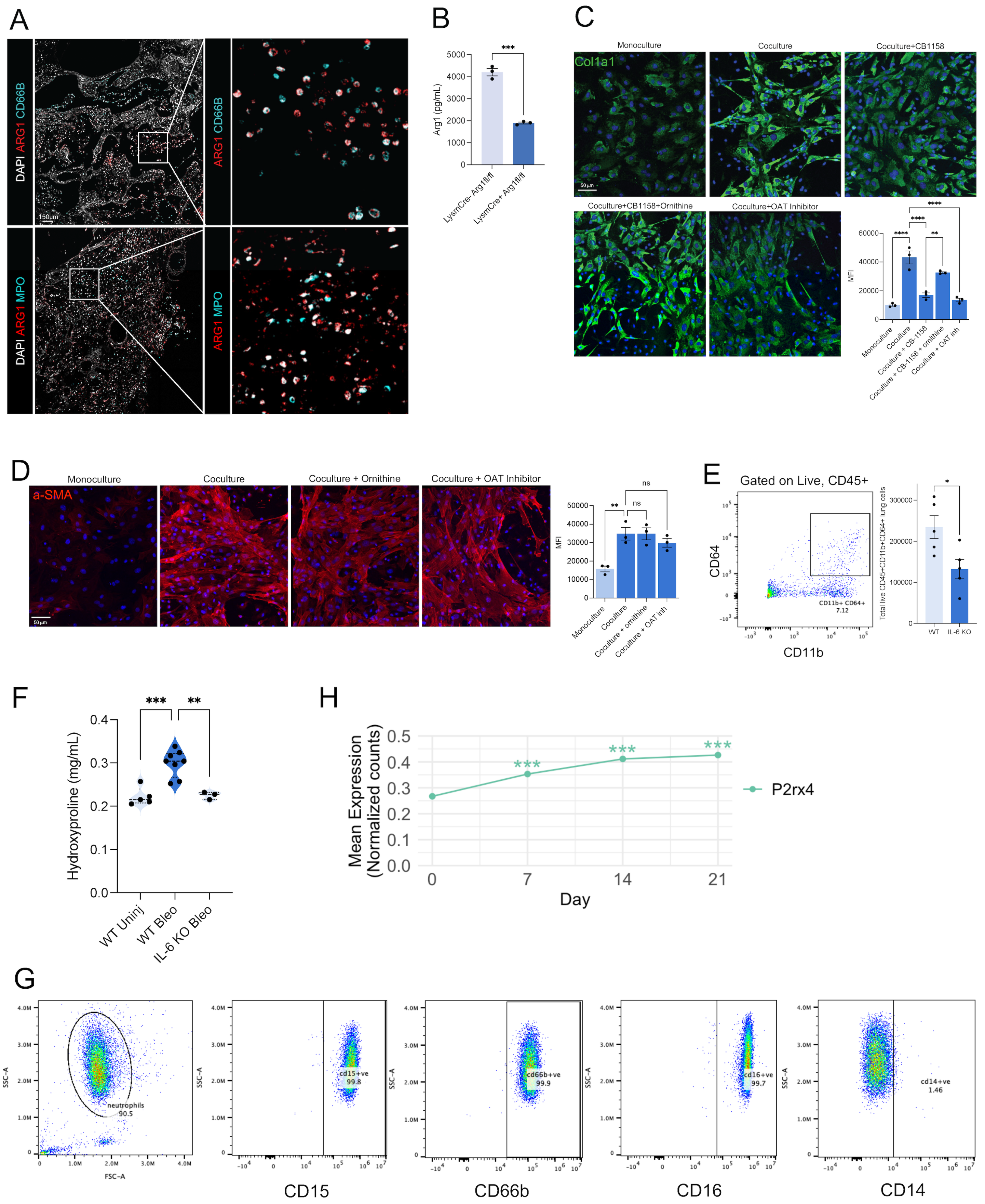

Supplemental Figure 3

A) Immunofluorescence of precision-cut lung slices (PCLS) from IPF lung. Displayed photomicrograph is representative of N=4 replicates.

B) ARG1 ELISA of lysates of CD11b+ cells freshly isolated by CD11b affinity column ***p<0.001 by Student's t test. Quantitation is for N=3 mice per condition. +/- SEM.
C) COL1A1 immunofluorescence of monocultured mouse lung fibroblasts with or without ornithine, ARG1 inhibitor CB-1158, or OAT inhibitor treatment. Quantification is for n=3 separate cultures each. **p<0.01, ****p<0.000 by 1-way ANOVA followed by post hoc Sidak's multiple comparisons tests. +/- SEM.
D) Alpha-smooth muscle actin immunofluorescence of monocultured mouse lung fibroblasts or murine lung macrophage-fibroblast cocultures, with or without ornithine or OAT inhibitor treatment. Quantification is for n=3 separate cultures each. **p<0.01 by 1-way ANOVA followed by post hoc Sidak's multiple comparisons tests. +/- SEM.
E) Representative FACS gating and quantitation of absolute lung cell number of live, CD45+CD11B+CD64+ cells in WT and IL-6 KO mice 14 days after bleomycin injury. Quantitation is for n=5 mice in each condition. *p<0.05 by Student's t test. +/- SEM.
F) Lung hydroxyproline for WT and *Il6* KO mice at 21 days after bleomycin injury. N=5, 8, 3 mice per condition, left to right. **p<0.01, ***p<0.001 by 1-way ANOVA followed by post hoc Sidak's multiple comparison's tests. +/- SEM.
G) Representative flow cytometry of neutrophils isolated from human donor peripheral blood. Individual markers are for the cells gated in the leftmost panel.
H) Expression of *P2rx4* across time after bleomycin injury in lung fibroblasts from Tsukui et al.(1) ***p<0.001.

References for Supplementary Figures

1. Tsukui T, Wolters PJ, and Sheppard D. Alveolar fibroblast lineage orchestrates lung inflammation and fibrosis. *Nature.* 2024;631(8021):627-34.
2. Strunz M, Simon LM, Ansari M, Kathiriya JJ, Angelidis I, Mayr CH, et al. Alveolar regeneration through a Krt8+ transitional stem cell state that persists in human lung fibrosis. *Nat Commun.* 2020;11(1):3559.
3. Fabre T, Barron AMS, Christensen SM, Asano S, Bound K, Lech MP, et al. Identification of a broadly fibrogenic macrophage subset induced by type 3 inflammation. *Sci Immunol.* 2023;8(82):eadd8945.
4. Aran D, Looney AP, Liu L, Wu E, Fong V, Hsu A, et al. Reference-based analysis of lung single-cell sequencing reveals a transitional profibrotic macrophage. *Nat Immunol.* 2019;20(2):163-72.
5. Li X, Mara AB, Musial SC, Kolling FW, Gibbings SL, Gerebtsov N, et al. Coordinated chemokine expression defines macrophage subsets across tissues. *Nat Immunol.* 2024;25(6):1110-22.
