# Supplementary material for: Myeloid-mesenchymal crosstalk drives ARG1-dependent profibrotic metabolism via ornithine in lung fibrosis
Source: J Clin Invest. 2025 Aug 28;135(21):e188734. doi: 10.1172/JCI188734 (PMC12578403; doi:10.1172/JCI188734)

Full unedited gels for Figure 3D (area outlined in green is shown in figure) for COL1A1 (top) and GAPDH (bottom).

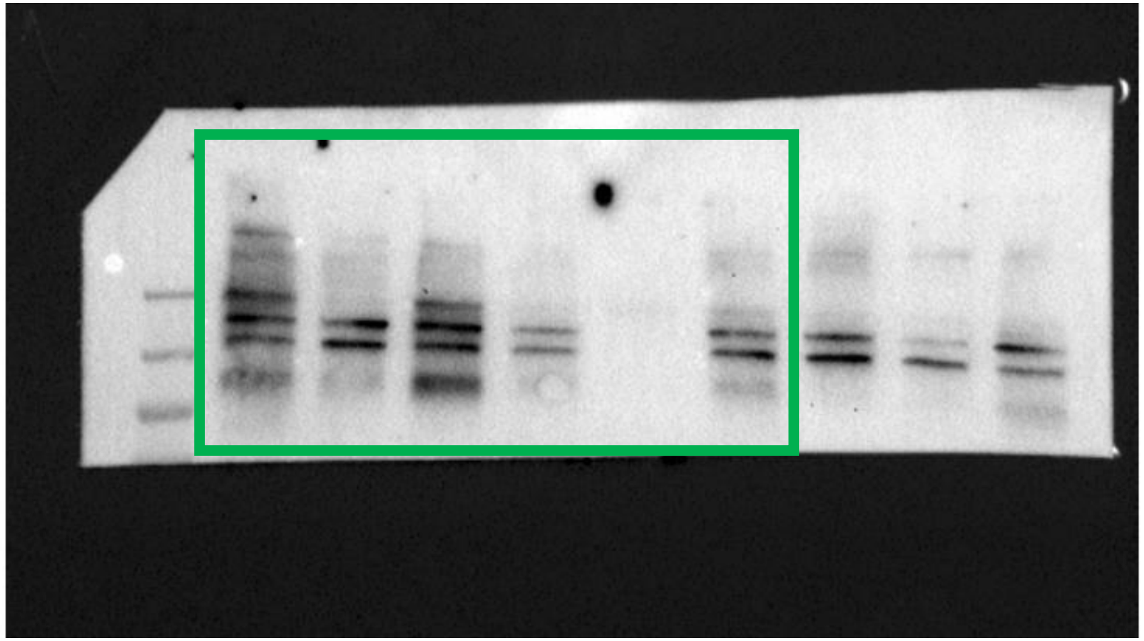

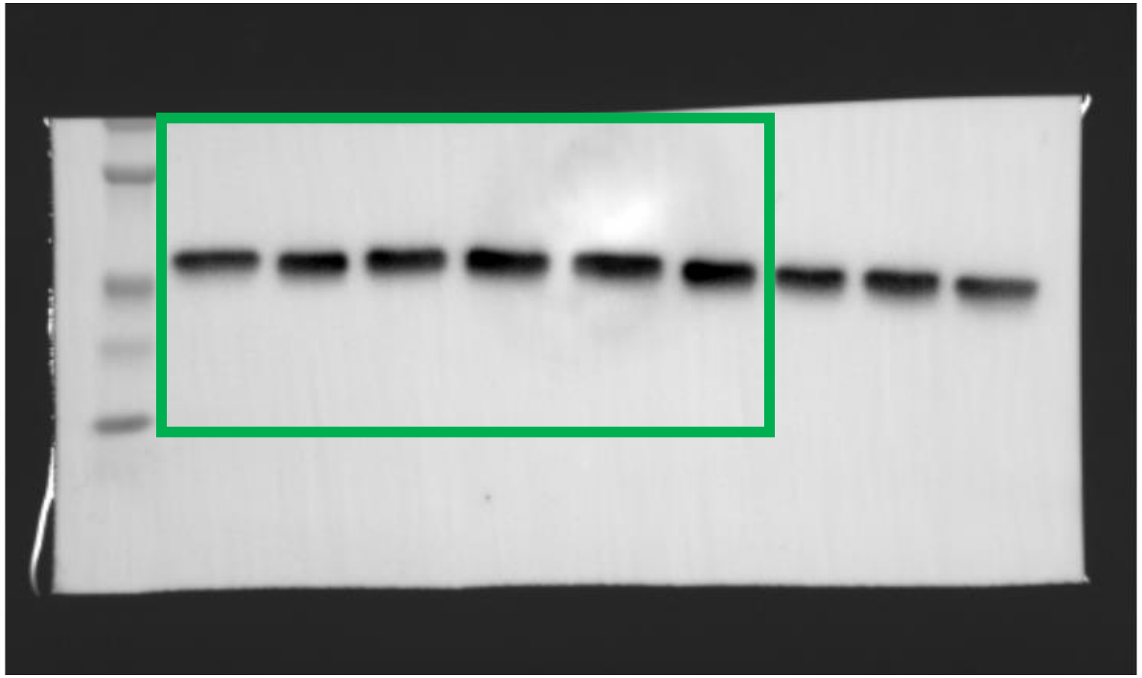

Supplement: Unedited blot and gel images [file jci-135-188734-s097.pdf]
